# Splicing of erythroid transcription factor is associated with therapeutic response in myelodysplastic syndromes

**DOI:** 10.1172/JCI189266

**Published:** 2025-05-27

**Authors:** Srinivas Aluri, Te Ling, Ellen Fraint, Samarpana Chakraborty, Kevin Zhang, Aarif Ahsan, Leah Kravets, Gowri Poigaialwar, Rongbao Zhao, Kith Pradhan, Anitria Cotton, Kimo Bachiashvili, Jung-In Yang, Anjali Budhathoki, Beamon Agarwal, Shanisha Gordon Mitchell, Milagros Carbajal, Srabani Sahu, Jacqueline Boultwood, Andrea Pellagatti, Ulrich Steidl, Amittha Wickrema, Satish Nandakumar, Aditi Shastri, Rajasekhar N.V.S. Suragani, Teresa V. Bowman, John D. Crispino, Sadanand Vodala, Amit Verma

**Affiliations:** 1 Department of Medicine, Albert Einstein College of Medicine, New York, New York, USA.; 2 Department of Hematology, St. Jude Children’s Research Hospital, Memphis, Tennessee, USA.; 3 Department of Developmental & Molecular Biology, Albert Einstein College of Medicine, New York, New York, USA.; 4 Bristol Myers Squibb, Summit, New Jersey, USA.; 5 Department of Cell Biology, Albert Einstein College of Medicine, New York, New York, USA.; 6 GenomeRxUS, Secane, Pennsylvania, USA.; 7 Radcliffe Department of Medicine, Oxford University, Oxford, United Kingdom.; 8 Department of Medicine, University of Chicago, Chicago, Illinois, USA.; 9 Bristol Myers Squibb, Hematology Translational Medicine, Summit, New Jersey, USA.

**Keywords:** Cell biology, Hematology, Hematopoietic stem cells

## Abstract

Anemia is the primary clinical manifestation of myelodysplastic syndromes (MDSs), but the molecular pathogenesis of ineffective erythropoiesis remains incompletely understood. Luspatercept, an activin receptor 2B (ACVRIIB-Fc) ligand trap, has been approved to treat anemia; however, its molecular mechanism of action is unclear. We found that activin receptor 2B (ACVR2B), its ligand growth and differentiation factor 11 (GDF11), and an effector, SMAD2, are upregulated in samples of patients with MDS. GDF11 inhibited human erythropoiesis in vitro and caused anemia in zebrafish, effects that were abrogated by luspatercept. Upon GDF11 stimulation of human erythroid progenitors, SMAD2 binding occurred in the erythroid regulatory regions, including at the GATA1 intron. Intronic SMAD2-binding led to skipping of exon 2 of *GATA1*, resulting in a shorter, hypomorphic isoform (*GATA1s*). CRISPR deletion of the SMAD2-binding intronic region decreased *GATA1s* production upon GDF11 stimulation. Expression of *GATA1s* in a mouse model led to anemia, rescued by a murine ActRIIB-Fc (RAP-536). Finally, RNA-Seq analysis of samples from the phase 3 MEDALIST trial revealed that responders to luspatercept had a higher proportion of *GATA1s* compared with nonresponders. Moreover, the increase in RBCs after treatment was linked to a relative decrease in *GATA1s* isoforms. Our study indicates that GDF11-mediated SMAD2 activation results in an increase in functionally impaired *GATA1* isoforms, consequently contributing to anemia and influencing responses to luspatercept in MDS.

## Abstract 2

We demonstrate that alternative splicing of GATA1 is seen in myelodysplastic syndrome and results in shorter functionally hypomorphic isoform. We demonstrate that GDF11 stimulated SMAD2 binding on GATAT1 intron is associated with GATA1-short and this is abrogated by Acitivin receptor 2B trap, Luspatercept.

## Graphical abstract

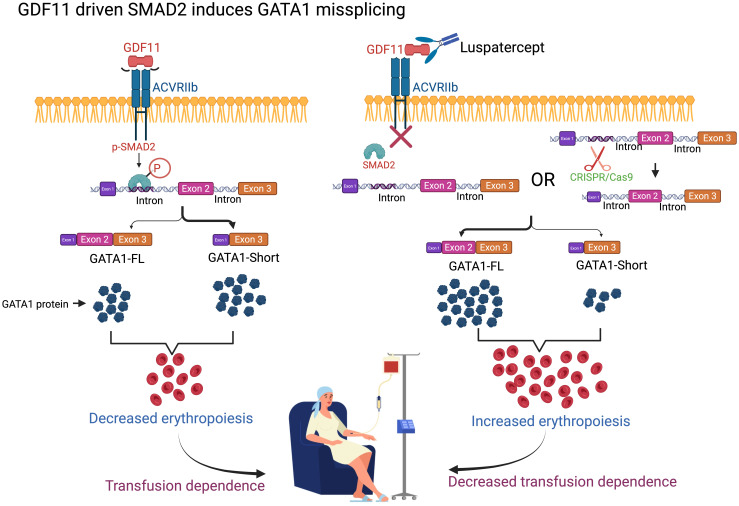

## Introduction

Myelodysplastic syndromes (MDSs) are a heterogeneous group of clonal stem cell disorders that result in ineffective erythropoiesis, leading to anemia as the major clinical manifestation (1). Mutations in RNA-splicing factors, epigenetic regulators, transcription factors, and oncogenes have been observed in MDS. However, the specific molecular mechanism that causes anemic symptoms is not well understood (2). Aberrant cytokine signaling is also postulated to contribute to defects in erythropoiesis, though most studies have focused on inflammatory cytokines (3–7). Anemia is the source of morbidity in most patients with MDS and requires further understanding of its underlying causes to drive advancements in treatment.

Luspatercept was recently approved by the FDA for the treatment of anemia in adult patients with lower-risk, transfusion-dependent MDS (8). Luspatercept is a fusion protein containing the activin receptor IIB fused with the Fc portion of human IgG (ActRIIB-Fc). It functions as a ligand trap for growth differentiation factors 8 and 11 (GDF11) as well as activin B, among other ligand proteins that belong to the TGF-β superfamily of ligands and transmit signaling through phospho-SMAD2/3. The MEDALIST and COMMANDS phase 3 clinical trials demonstrated that luspatercept treatment resulted in significant increases in RBC production in patients with MDS (8, 9). However, the precise mechanism by which luspatercept restores erythropoiesis in patients with MDS remains to be investigated further. The role of GDF11 has been studied in skeletal muscle regeneration and aging, but its role in regulating human hematopoiesis has not been well defined (10–12). We currently lack an understanding of the downstream signaling pathways triggered by GDF11 in human hematopoietic stem cells (HSCs).

Here, we show that GDF11, its cognate receptor ACVR2B, and its downstream effector SMAD2 are overexpressed in MDS samples. GDF11-driven SMAD2 binds to the first intron of *GATA1* and induces the expression of a shorter and functionally deficient isoform of *GATA1* in erythroid progenitors. CRISPR deletion of the intron binding region of *GATA1* reverses the effects of SMAD2-dependent short-isoform production. GDF11 treatment induces anemia in the zebrafish model, which could be rescued by luspatercept. Expression of the *GATA1* short isoform (*GATA1s*) in a mouse model led to anemia, which was rescued by the murine version of luspatercept, RAP-536 (human ActRIIB fused with murine Fc). Last, we demonstrate that, in the MEDALIST study, patients who responded to luspatercept expressed a relatively higher proportion of the short isoform of *GATA1* when compared with nonresponders at baseline. Interestingly, after 24 weeks of luspatercept treatment, increases in RBC production were associated with an increase in the ratio of full-length *GATA1* to *GATA1s*.

## Results

### GDF11 and ACVR2B are overexpressed in MDS.

**Figure 1:**
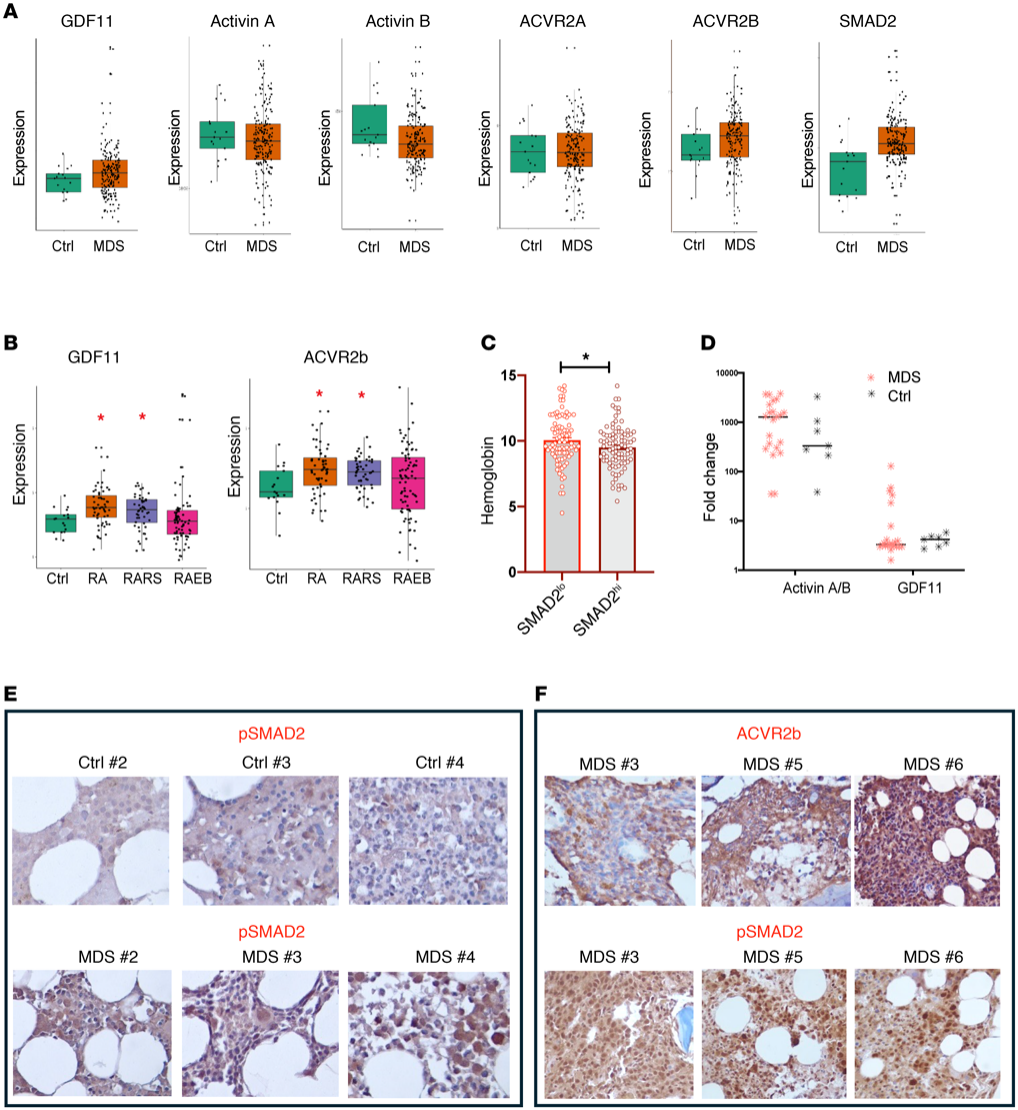
GDF11-SMAD2 signaling is upregulated in samples from patients with MDS. (**A**) Comparison of RNA expression of GDF11, activin A, activin B, ACVR2A, ACVR2B, and SMAD2 in samples from 183 patients with MDS and age-matched healthy control (Ctrl) participants. (**B**) RNA expression of GDF11 and ACVR2B between age-matched controls and patients with refractory anemia (RA), refractory anemia with ring sideroblasts (RARS), and refractory anemia with excess blasts (RAEB). (**C**) Comparison of hemoglobin levels in patients with low and high SMAD2 expression. (**D**) Analysis of cytokines (GDF11 and activin A/B) in serum samples from patients with MDS and healthy control participants. (**E** and **F**) Immunohistochemistry analysis of bone marrow samples from patients with MDS to assess pSMAD2 and ACVR2B expression. *P < 0.05, Student’s *t* test.

We analyzed the expression of components of the activin/GDF/ActRII pathway in CD34^+^ cells from 183 patients with MDS and 17 age-matched control participants (13). We found significantly higher expression of *GDF11, ACVR2B,* and *SMAD2* mRNA in MDS hematopoietic stem and progenitor samples compared with those of the age-matched control participants (Figure 1A). Notably, patient samples of the lower-risk MDS subtypes refractory anemia and refractory anemia with ring sideroblasts, in which anemia is the predominant clinical presentation, have significantly elevated levels of *GDF11* and *ACVR2B* transcripts (Figure 1B). Additionally, we observed that samples with higher SMAD2 expression had significantly lower hemoglobin (Hb) values (Figure 1C). Our results show detectable levels of GDF11 in the peripheral blood plasma of a subset of patients with MDS, indicating a potential role for GDF11 in MDS pathophysiology (Figure 1D). Immunohistochemical examinations have demonstrated increased levels of phosphorylated or activated SMAD2 (pSMAD2) in bone marrow samples from patients with elevated GDF11 levels, as well as detectable ActRIIB expression in MDS bone marrow samples (Figure 1, E and F).

### GDF11 induces ineffective erythropoiesis.

**Figure 2:**
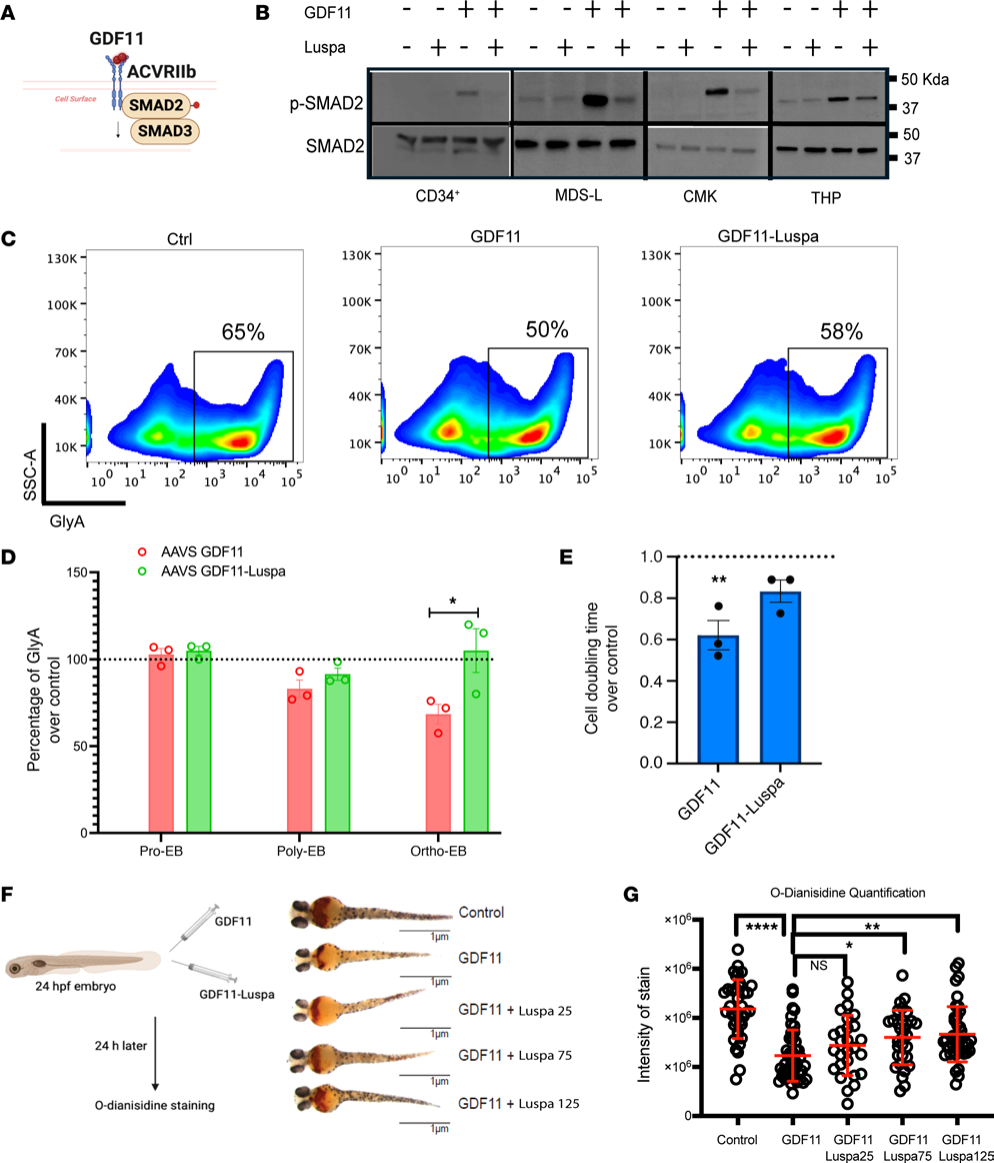
GDF11 induces ineffective erythropoiesis in zebrafish and HSC culture. (**A**) The schematic illustrates GDF11 binding to the ActRIIb receptor and activating SMAD2 through phosphorylation (marked with a red dot). (**B**) The Western blot displays pSMAD2 and SMAD2 expression in various cell lines, including HSCs (d6 erythroid progenitors), MDS-L, CMK, and THP1. The symbols – and + denote the absence or presence, respectively, of specific ligands or drugs. This image represents findings from 3 independent experiments. (**C**) The flow plots exhibit the emergence of glycophorin A (GlyA)-positive colonies after treatment with the GDF11 and GDF11- luspatercept (Luspa) combinations. Population percentages are indicated. SSC-A, side scatter (area). (**D**) GlyA population measurements from 2 independent experiments at different late erythroid differentiation stages are plotted under control (Ctrl), GDF11, and GDF11-Luspa conditions (*n* = 3). Pro-EB, proerythroblast; Ortho-EB, orthochromatic erythroblast; poly-EB: ploychromatic erythroblast. *P < 0.05, Student’s *t* test. (**E**) Bar plots demonstrate an increase in cell doubling times for HSCs treated with GDF11 and a decrease in cell doubling times for the GDF11-Luspa combination (*n =* 3). *P < 0.05, Student’s *t* test. (**F**) The diagram depicts the injection of GDF11 or Luspa into zebrafish larvae. Images demonstrate the hemoglobinization of zebrafish larvae after injections of GDF11 or Luspa. (**G**) *O*-Diansidine quantification data from 3 independent experiments are depicted. *P < 0.05, **P < 0.005, ***P < 0.001, ****P < 0.0001, ANOVA.

Next, we wanted to determine the functional role of GDF11 in hematopoiesis. We demonstrate that GDF11 phosphorylates SMAD2 in primary human CD34^+^ hematopoietic stem and progenitor cells (HSPCs) and myeloid cell lines, which is abrogated by luspatercept treatment (Figure 2, A and B). To determine the functional effects of GDF11 on human erythropoiesis, we generated erythroid precursors from primary human CD34^+^ HSPCs using an erythroid differentiation cocktail (14). We then treated progenitors in the burst forming unit-erythroid (BFU-E)/CFU-E stage with GDF11 with and without luspatercept. We found that exposure to GDF11 resulted in a reduction in glycophorin A–positive cells toward the later stages of erythroid maturation (Figure 2, C and D). We calculated cell doubling times with GDF11 treatment to assess its impact on cell division. Our analysis showed that GDF11 exposure decreases cell doubling times during erythroid differentiation (Figure 2E). Both effects were rescued upon treatment with luspatercept, suggesting an inhibitory role for GDF11 in later stages of erythroid differentiation.

Last, we tested the effects of GDF11 in vivo in zebrafish. Treatment of zebrafish embryos with exogenous GDF11 resulted in a decrease in hemoglobinization when compared with controls (Figure 2F). Treatment with luspatercept resulted in the rescue of anemic phenotypes in zebrafish in a dose-dependent fashion (Figure 2G).

### GDF11/SMAD2 drives production of a hypomorphic GATA1 isoform.

**Figure 3:**
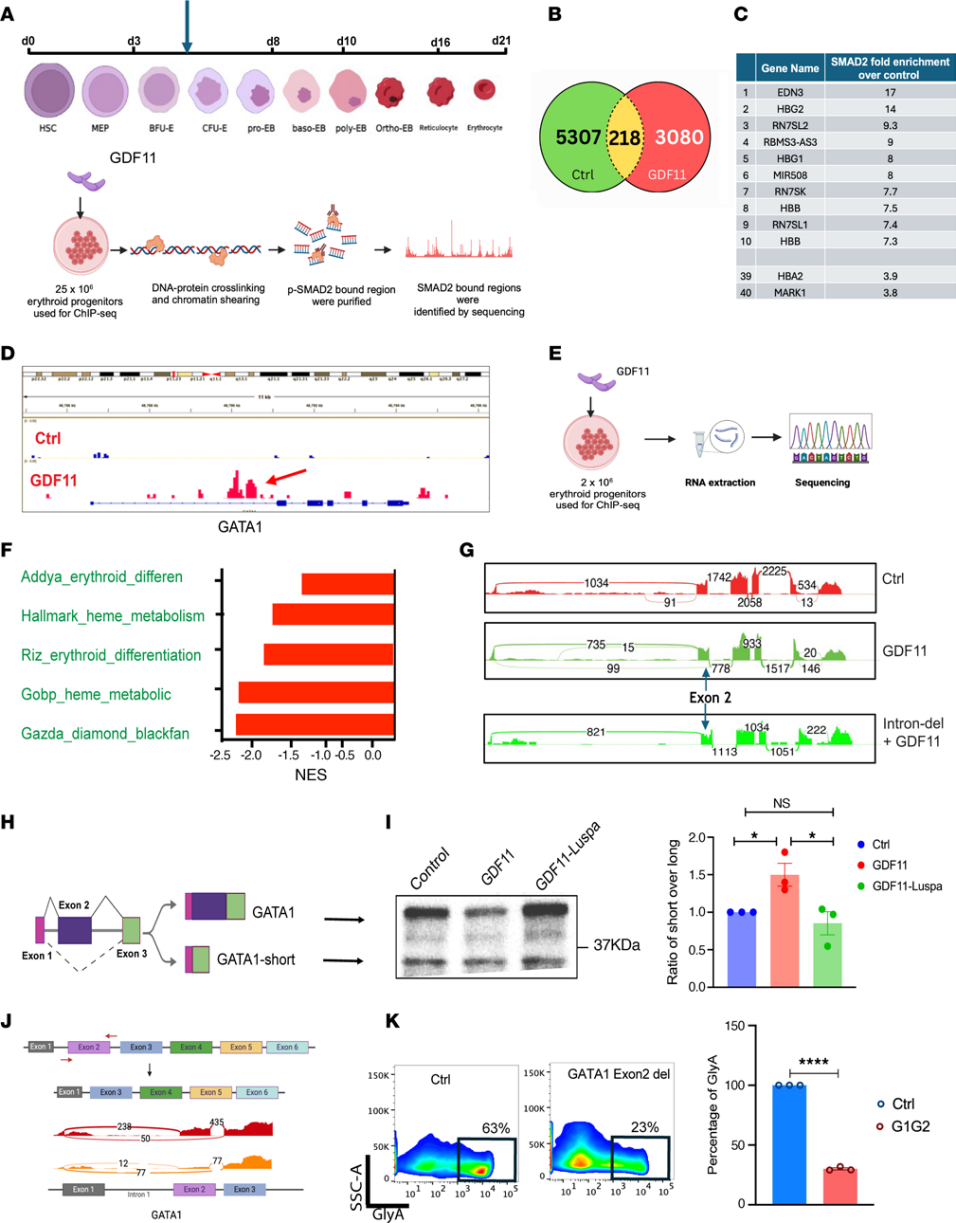
GDF11-driven pSMAD2 binds to the intronic region of *GATA1*. (**A**) Schematic showing HSC differentiation and cell stages across different days. On day 5, approximately 25 million erythroid progenitors were treated with GDF11; chromatin immunoprecipitation was carried out, followed by the sequencing of pSMAD2 pull-down peaks. (**B**) Venn diagram showing the number of peaks that are found in both control (Ctrl) and GDF11 and in individual samples. (**C**) List of genes that are top hits in the pSMAD2 ChIP. (**D**) Integrative genomics viewer (IGV) tracks showing pSMAD2 binding in the *GATA1* intron in both control and GDF11-treated samples. The arrow indicates the pSMAD2 binding peak. Both tracks are normalized for input. (**E**) Schematic of RNA preparation from erythroid progenitors upon GDF11 stimulation. (**F**) Pathway analysis for erythroid-related pathways was conducted through gene set enrichment analysis and highlights the downregulated pathways. NES, normalized enrichment score. (**G** and **H**) A schematic representation of the alternative splicing of *GATA1* into *GATA1* and *GATA1*-short. (**I**) The Western blot image demonstrates that GDF11 treatment reduces the overall expression of both *GATA1* full-length and *GATA1s*, resulting in an increased short to long isoform ratio. The effects of GDF11 can be attenuated by the addition of luspatercept (left). A bar plot displays the *GATA1s* to *GATA1* long isoform ratio from 3 independent experiments conducted on erythroid progenitors (right). *P < 0.05, ANOVA. (**J**) The schematic illustrates the strategy for deleting *GATA1* exon 2 using CRISPR/Cas9. The top image depicts the WT *GATA1*, and the bottom sashimi plot shows the *GATA1* with exon 2 deleted (del). RNA-Seq verified the reduction in *GATA1* exon 2. (**K**) A flow plot demonstrates the emergence of glycophorin A (GlyA)-positive cells after *GATA1* exon 2 deletion, with indicated population percentages (left). A bar plot showing the average of 3 independent experiments (right). (**J**). *P < 0.05, Student’s *t* test.

Next, we examined downstream effects mediated by GDF11 in erythropoiesis by treating primary human erythroid progenitors at the BFU-E/CFU-E stage of maturation with 100 ng/mL GDF11 or vehicle control (Figure 3A). To assess pSMAD2 binding on the genome and analyze GDF11/pSMAD2-induced transcriptomic changes, we performed ChIP-Seq and RNA-Seq on the same samples. We observed 5307 unique SMAD2 binding peaks in the control sample and 3080 in GDF11-treated sample (*q* = 0.01) (Figure 3B). We observed enrichment of SMAD2 binding in GDF11-treated samples in gene loci involved in erythropoiesis, including numerous hemoglobin genes and transcription factors (Figure 3C and Supplemental Figure 1A; supplemental material available online with this article; https://doi.org/10.1172/JCI189266DS1). Interestingly, upon GDF11 treatment, we observed SMAD2 binding in the intronic region of the master erythroid transcription factor, *GATA1*. (Figure 3D). We further confirmed the SMAD2 binding at *GATA1* intron 1 by ChIP-qPCR (Supplemental Figure 1B). GDF11 administration also led to numerous transcriptomic changes in parallel RNA-Seq assays. Pathway analysis revealed that hemoglobin and Myc-regulated pathways are specifically downregulated (Figure 3F and Supplemental Figure 2A) upon GDF11 stimulation. The transcriptional levels of *GATA1* were also decreased, as indicated by our qRT-PCR analysis of *GATA1* mRNA (Supplemental Figure 2B). A more detailed analysis of *GATA1* expression at the exon level revealed an increase in exon 2 skipping after GDF11 stimulation (Figure 3G).

To determine the functional role of the intronic SMAD2 binding region in exon 2 skipping, we used CRISPR/Cas9 to delete a 700 bp region (Supplemental Figure 3A) in the intron 1 of primary human erythroid progenitor cells. We chose this region also because it harbors the SMAD2 binding motif (15) (Supplemental Figure 3C). We then analyzed the exon-level expression of *GATA1* transcripts after GDF11 treatment. The CRISPR/Cas9 deletion showed a deletion efficiency of about 50%, by qPCR (Supplemental Figure 3B). We also observed that deletion of the pSMAD2 binding intronic element prevented the exon skipping event that is directed by GDF11 (Figure 3G, lower panel).

Exon 2 skipping in the *GATA1* gene leads to a transcript that encodes a shorter isoform of *GATA1*, *GATA1s*, that is hypomorphic in function (16) (Figure 3H). Immunoblotting confirmed that a greater relative proportion of *GATA1s* was produced in human primary erythroid progenitors upon GDF11 stimulation; this was reversed after treatment with luspatercept (Figure 3I).

Next, to determine the functional role of *GATA1s* in primary human erythropoiesis, we used CRISPR/Cas9 to delete the second exon of the *GATA1* gene in primary human CD34^+^ HSPCs (17). We achieved 55% CRISPR deletion efficiency through this method (Supplemental Figure 3A). In CRISPR-deleted cells, the second exon transcript was significantly less, and *GATA1s* was relatively overrepresented (Figure 3J). Immunoblotting confirmed the relative shift from the long to the short isoform of *GATA1* upon exon 2 deletion at the protein level (Supplemental Figure 3B). These cells were differentiated, and erythropoietic efficiency was diminished compared with controls. The number of glycophorin A–positive cells decreased significantly upon exon 2 deletion. (Figure 3K). These findings indicate that primary erythroid cells exposed to GDF11 express a relative increase in the shorter hypomorphic *GATA1* isoform, which is less effective at promoting erythropoiesis.

### The ActRIIB ligand trap can ameliorate anemia in mice, with enforced expression of the GATA1s isoform.

**Figure 4:**
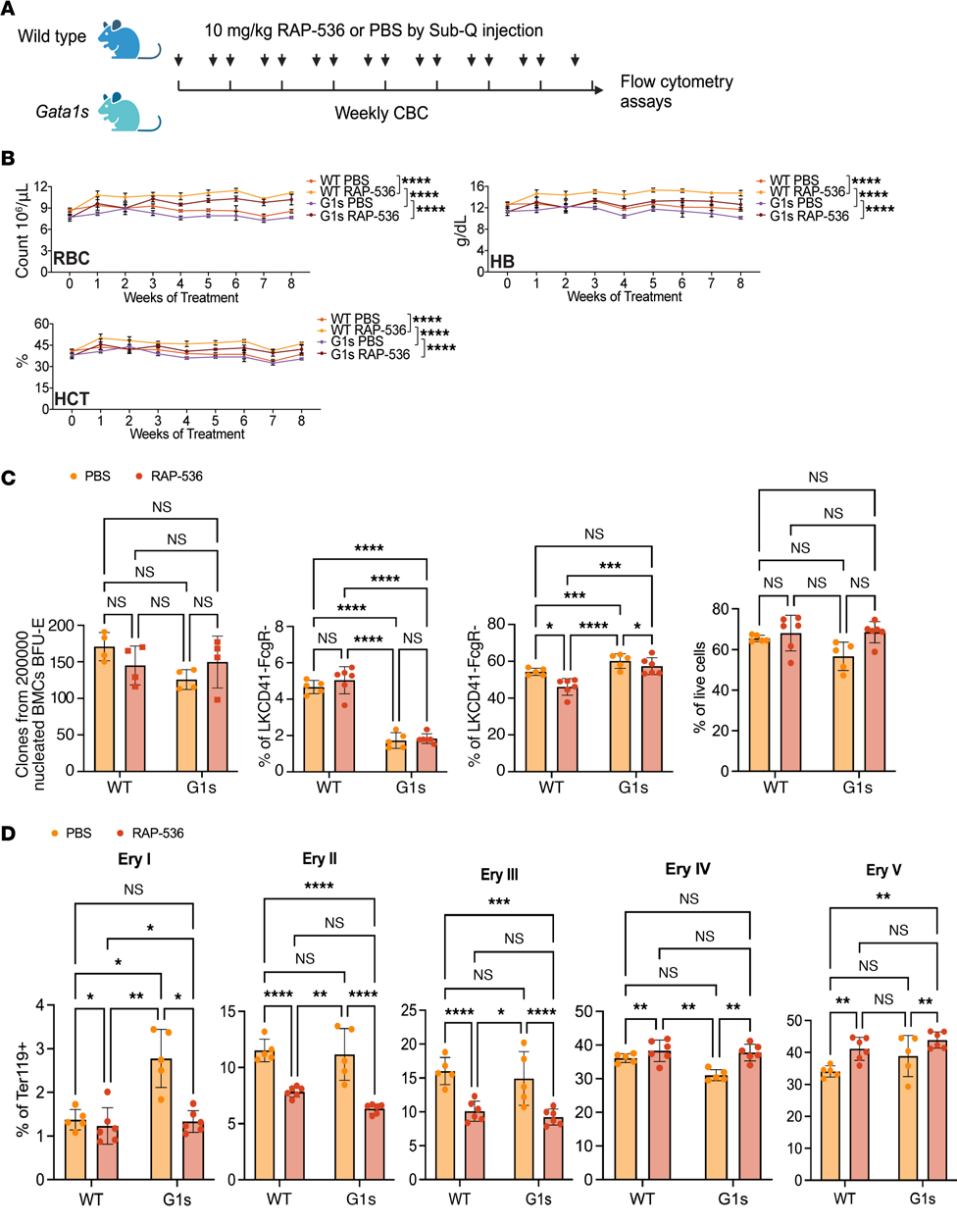
Mouse version of luspatercept rescue anemia in *gata1s* mouse. (**A**) Experimental schema. Male *Gata1s* and WT littermates were treated with RAP-536 for 8 weeks and were bled to determine the CBC count weekly. At the end of the study, mice were euthanized and detailed analysis conducted of hematopoiesis in the bone marrow and progenitor numbers. (**B**) RBC indices at baseline and during the 8 weeks of treatment. ****P < 0.0001, 2-way ANOVA. (**C**) Far left, BFU-e colony-forming assays; the next 3 panels show the percentages of pre-CFUe, CFUe and proE, and Ter119^+^ cell populations in the bone marrow. Each dot depicts an individual mouse. *P < 0.05, **P < 0.01, ***P < 0.001, 2-way ANOVA. (**D**) Percentages of the 5 different erythroid populations in the bone marrow. Each dot depicts an individual mouse. MCV, mean corpuscular volume. **P < 0.01; ***P < 0.001; ****P < 0.0001, 2-way ANOVA. Sub-Q, subcutaneous.

A murine model for determining the functional in vivo role of *GATA1s* was engineered by targeted deletion of exon 2 (18). These mice exhibit lifelong macrocytic anemia with decreases in RBC count, Hb, and hematocrit (HCT), and an increase in mean corpuscular volume (19). We treated *gata1s* mice and WT littermates with RAP-536 (the murine version of luspatercept) for 8 weeks (twice per week, i.p.) (Figure 4A). RAP-536 administration led to an increase in RBC count, Hb, and HCT in the mouse cohorts (Figure 4B). In addition, RAP-536 treatment led to an increase in bone marrow erythroid progenitors (populations I, II, and III) and levels of BFU-E, pre-CFUe, and Ter119^+^ erythroid cells when compared with controls (Figure 4C). RAP-536 also increased later stages of bone marrow erythroblast differentiation (Ery I to V) (Figure 4D). Analysis of GATA1s expression in Ter119^+^ cells showed an increase in GATA1s protein expression after RAP-536 treatment (Supplemental Figure 4). Consistent with published studies that showed overexpression of Gata1s can rescue anemia caused by the absence of GATA1 (20), we surmise that the increased expression of GATA1s is sufficient to overcome the hypomorphic activity of the Gata1s isoform, accounting for the rescue of ineffective erythropoiesis.

Next, we conducted a mixed chimera study to examine the impact of RAP-536 on erythroid cell production, transplanting equal numbers of total bone marrow cells from male *Gata1s* and WT competitors into irradiated recipient mice (Supplemental Figure 5A). RAP536 increased the RBC count, Hb, and HCT in the peripheral blood of the recipients (Supplemental Figure 5B). RAP-536 also led to an expansion of WT PBMCs over *Gata1s* and an increased proportion of the mature Ery II, III, and IV populations in the bone marrow by the end of the study (Supplemental Figure 5, C and D). The earlier erythroid progenitor populations, which are preMegE, pre-CFUe, CFUe/proE, and Ery I, did not change upon treatment, again demonstrating the effects of ActRIIB inhibition on later stages of erythropoiesis (21–23) (Supplemental Figure 5E).

### Alternatively spliced GATA1 short isoform is associated with clinical responses to luspatercept.

**Figure 5:**
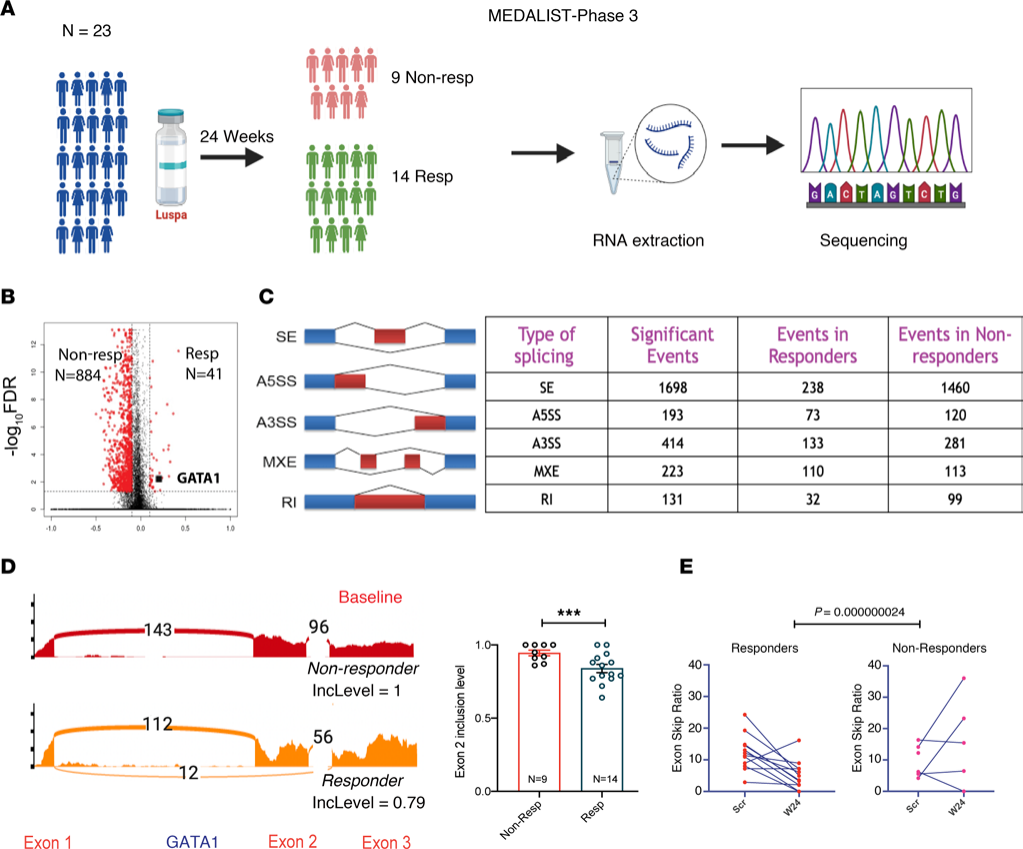
Responders to luspatercept have *GATA1s* isoforms. (**A**) The phase 3 MEDALIST trial involved 24 patients, with 9 classified as nonresponders and 14 as responders. Total RNA was extracted from all patient samples and analyzed through RNA-Seq for further investigation. (**B**) A volcano plot displayed the total number of splicing events in both responder and nonresponder groups. *GATA1* is indicated. (**C**) A schematic illustrates various types of RNA splicing. An rMATS analysis summarized the splicing events observed in both groups. (**D**) Representative sashimi plot images depict *GATA1* splicing in nonresponders (top) and responders (bottom), showcasing exon inclusion levels (IncLevel). The organization of *GATA1* exons was presented beneath the sashimi plot (left). A bar plot demonstrated the *GATA1* exon 2 inclusion in 9 nonresponders (non-Resp) and 14 responders (Resp) at baseline in the MEDALIST trial, with *P* values adjusted for multiple comparisons (right). ***P < 0.005. (**E**) Line plots illustrate the *GATA1* exon 2 skip ratio for responders and nonresponders at screening (Scr) and week 24 (W24). *P < 0.00000002.

Finally, we investigated whether there are any associations between *GATA1* splicing and therapeutic response in patients with MDS. The phase 3 MEDALIST trial showed that treatment with luspatercept resulted in RBC transfusion independence in 40% of patients with MDS, though no molecular predictors of response were identified (8). We performed RNA-Seq on pre- and post-treatment bone marrow samples from 23 trial participants (Figure 5A). Pretreatment samples from responders and nonresponders were not associated with any significant differentially expressed genes by global transcriptomic analysis. By contrast, examination of RNA splicing showed significant differences between responders and nonresponders, with nonresponders having a higher number of alternative splicing events (Figure 5, B and C, and Supplemental Table 1). Despite fewer global splicing events, at baseline or before treatment, responders to luspatercept had a significant increase in *GATA1* exon 2 skipping events in bone marrow samples (Figure 5D). After 24 weeks of luspatercept treatment, responders had reduced relative exon 2 skipping events in *GATA1* transcripts (Figure 5E), and an interaction model showed a significant difference (*P =* 2.42 × 10^–8^) in the *GATA1* to *GATA1s* ratio between the responders and nonresponders. These findings demonstrate that the response to luspatercept is correlated with the relative presence of *GATA1s* in MDS.

## Discussion

Myelodysplastic syndromes are a heterogeneous group of diseases characterized by cytopenias, with anemia as the predominant clinical abnormality. Previous studies established that GDF11 negatively regulates erythropoiesis in murine models, though the underlying mechanisms remained unclear (22). Suragani et al. further demonstrated that GDF11 impairs terminal erythroid maturation, but they did not elucidate a downstream molecular mechanism for this effect (22). Our study shows that GDF11 signaling affects erythroid differentiation by altering *GATA1* splicing. *GATA1* is a master regulator of erythropoiesis. Its expression is tightly regulated, and mutations or cleavage by caspases cause overt anemia or dyserythropoiesis in various experimental models (24–26). Specifically, mutations at the splicing junction of the second exon result in the exclusive production of the short isoform of the *GATA1*, which is a less active form of the protein. Intronic mutations affecting *GATA1* splicing occur in rare pediatric anemias (27).

The role of *GATA1s* in MDS remains unexplored. Previous studies reported *GATA1s* is a poor prognostic factor. Furthermore, *GATA1s* overexpression altered complex II activity of the electron transport chain by modulating succinate dehydrogenase and limiting oxidative phosphorylation. *GATA1s* was also reported to prevent ferroptosis in K562 cell lines, indicating a pro-leukemogenic phenotype (28–30). This study is the first, to our knowledge, to demonstrate that the ratio of *GATA1* to *GATA1s* is reduced in certain subsets of patients with MDS, and these patients respond positively to treatment with luspatercept. Most patients in this trial carried mutations in the splicing factor *SF3B1* within their HSCs, with a few exceptions. Thus, our analysis reveals no clear correlation between the presence of splicing factor mutations and the occurrence of the GATA1 short isoform, suggesting that alternative mechanisms may be driving its splicing regulation. We propose that a mechanism behind the rescue of erythropoiesis by luspatercept is through the GDF11/pSMAD2/*GATA1* axis, whereby GDF11-driven SMAD2 binds to the intronic region of *GATA1*, inducing the production of *GATA1s*. Furthermore, our study demonstrates that the deletion of the *GATA1* intron using CRISPR/Cas9 led to a reduced expression of *GATA1s* after GDF11 treatment.

The TGF superfamily comprises many ligands that play important roles in the regulation of hematopoiesis. These ligands stimulate the SMAD transcription factors and drive changes in gene expression. Normal erythropoiesis depends on the precise control of pSMAD2 signaling. Dysregulation of this signaling system in MDS impairs erythropoiesis (31). Although ALK5 inhibitors have demonstrated potential in preclinical studies to target the TGF-β/SMAD axis, these efforts have not resulted in significant therapeutic improvements (32). However, ligand traps targeting TGF-β superfamily cytokines have seen significant clinical success (33, 34). Luspatercept, which traps GDF11, has demonstrated success in restoring normal erythropoiesis and reducing the need for frequent blood transfusions in patients (8, 9). Studies done in mice with β-thalassemia showed that RAP-536, the mouse version of luspatercept, increases nuclear levels of *GATA1* and an increase in the *GATA1* gene signature; however, the precise mechanism was not known (35). Our results demonstrate the important novel regulatory roles of GDF11 overexpression in human erythropoiesis models and *GATA1* isoform expression.

## Methods

### Sex as a biological variant

In this study, sex was not explicitly considered a biological variable for human and animal samples. The mechanisms investigated are not known to be sex specific, and our analyses were not stratified by sex.

### Animal models and cell lines

#### Zebrafish.

Zebrafish were maintained as described by Lawrence et al (36).

#### gata1 Short mouse.

The *gata1*^Δex2^ mice (referred to as *Gata1s* or G1s), which carry a deletion of exon 2 causing unique expression of GATA1s, were provided by Stuart Orkin from Children’s Hospital in Boston, Massachusetts, USA.

### Patient samples

Primary samples were obtained from patients diagnosed with MDS. Human HSCs were obtained by isolating CD34^+^ cells from allo donors. Cells lines such as, K562, THP1, CMK, KG1a, and MV-4-11 were obtained from ATCC and cultured in IMDM supplemented with 10% FBS and 1% penicillin-streptomycin (pen-strep). MDS-L was provided by Kaoru Tohyama (Kawasaki Medical School, Okayama, Japan). MEDALIST trial samples were obtained from Bristol Myers Squibb.

### Bacterial strains

Plasmid transformations were conducted in XL-10 gold competent cells from Agilent. Transformants were either selected on ampicillin or kanamycin.

### RNA isolation and qRT-PCR

The cells were initially pelleted at 350*g* and then resuspended in an RLT buffer containing 1% β-ME, which helped stabilize the RNA. The cell pellets were thoroughly resuspended to ensure complete lysis and stored at –80°C until further processing. To isolate the total RNA, the Qiagen RNeasy kit was used following the manufacturer’s protocol. The purified RNA was quantified using a Qubit instrument to determine its concentration. Subsequently, 1000 ng of RNA was reverse transcribed into cDNA using a SuperScript IV VILO master mix according to the manufacturer’s instructions. The resulting cDNA was diluted 10-fold, and 1 μL was used as a template for qPCR analysis. For qPCR amplification, EvaGreen Supermix was used. The fold change in expression was determined by the comparative Ct method, and statistical significance was assessed using an unpaired Student’s *t* test. The MEDALIST trial samples were already received in the RLT lysis buffer. We processed them using the QIAgen All Prep Kit, which allows for the isolation of RNA, genomic DNA, and protein samples from a single sample that is resuspended in RLT. The RNA was isolated following the manufacturer’s protocol and quantified using a Qubit instrument.

### RNA library preparation

The RNA quality was analyzed using a bioanalyzer. RNA samples with RNA quality number scores higher than 8 were considered suitable for RNA library preparation. The library preparation process followed the manufacturer’s protocol (QIAseq Stranded RNA Lib Kit UDI-B; QIAGEN, 180452). Initially, RNA molecules were fragmented into smaller fragments, and then reverse transcriptase was used to convert the fragmented RNA into double-stranded cDNA. Adaptor ligation was performed, followed by library amplification to ensure sufficient cDNA fragments for sequencing. Prior to sequencing, the library underwent quality control and quantification. Sequencing was conducted using an Illumina sequencer (Nextseq500) with 150 paired-end sequencing, resulting in the generation of approximately 30 million reads per sample.

### RNA-Seq read mapping, differential expression analysis, and splicing analysis

We used HISAT2 2.0.0-beta to align readings with GRCh38. The htseq-count tool was used to find the total number of read pairs that were uniquely mapped. We have made the data accessible through the NCBI’s Gene Expression Omnibus (GEO) repository. To identify transcripts undergoing alternative splicing (AS) events between conditions, we used the Robust Multi-array Average Transcript Splicing (rMATS) tool. This tool also was used to detect various AS events, such as skipped exons, alternative 5′ or 3′ splice sites, and retained introns. The inclusion level (percent spliced-in [PSI]) for each splicing event across samples was calculated. Using rMATS, a statistical model was applied to pinpoint AS events showing significant changes in PSI between conditions. Differentially spliced events were identified based on a user-defined fold-change threshold for PSI alteration and an adjusted *P* value threshold for statistical significance.

### ChIP-Seq methodology

A total of 25 million cells were treated with GDF11 and TGF-β1. After a 2-hour treatment, the cells were pelleted at 350*g* for 15 minutes at 4°C. They were then washed once with ice-cold PBS. The following steps were followed using the HighCell ChIP Kit (Diagenode, C01010060), according to the manufacturer’s protocol. To begin fixation, 13.5 μL of 36.5% formaldehyde was added per 500 μL of sample, achieving a final concentration of approximately 1%. The mixture was gently vortexed and incubated for 8 minutes at room temperature. To stop the fixation process, 57 μL of 1.25 M glycine was added, gently mixed by vortexing, and incubated for 5 minutes at room temperature. The sample was then centrifuged at 1500 rpm (300*g*) for 5 minutes at 4°C. The supernatant was carefully discarded, leaving approximately 30 μL of solution behind to avoid disturbing the cross-linked cells. The cross-linked cells were washed twice with 1 mL of ice-cold PBS, leaving about 10–20 μL behind after the final wash. Next, 1 mL of ice-cold lysis buffer L1 was added per pellet of cells and resuspended by pipetting. The mixture was incubated for 10 minutes at 4°C with gentle mixing. Centrifugation for 5 minutes at 1600 rpm (500*g*) at 4°C followed, and the supernatant was discarded. The lysis process was repeated with 1 mL of ice-cold lysis buffer L2.

The complete shearing buffer S1 for sonication was made by adding a protease inhibitor and centrifuging again. The supernatant was then thrown away. Then, 200 μL of the buffer was added to the cells, followed by vortexing to resuspend and incubation for 10 minutes on ice. The chromatin was sheared using a Bioruptor, following appropriate shearing conditions based on the cell type and system used. We added 5 μL of protease inhibitor mix per 1 mL of ChIP buffer C1. For IP, 800 μL of ChIP buffer C1 was mixed with 200 μL of sheared chromatin. The mixture was then spun at 12,000 rpm (10,000*g*) for 10 minutes to obtain the supernatant.

The antibody-coated beads were prepared by briefly spinning them and placing them in an ice-cold magnetic rack. The supernatant was discarded, and the pellet of beads was retained. Then, 950 μL of diluted sheared chromatin was transferred to each IP tube, with 9.5 μL kept as an input sample at 4°C. The tubes were incubated under constant rotation at 4°C overnight. On the following day, the tubes were spun and placed in the magnetic rack to discard the supernatant. The beads were washed 3 times with ice-cold ChIP buffer C1 and once with buffer W1, following the specified washing procedures. The input samples were centrifuged briefly and treated in parallel with the IP samples from this point on. Proteinase K was added to the DNA isolation buffer to make the full buffer. An appropriate amount of this buffer was then added to both the immunoprecipitated DNA samples and the input DNA samples. After 15 minutes at 55°C, all samples were incubated for 15 minutes at 100°C. The tubes were then briefly spun, placed in the ice-cold magnetic rack, and the supernatants were collected for sequencing or qRT-PCR.

### ChIP-Seq analysis and peak calling

After the ChIP-Seq library preparation and sequencing, we used the Model-based Analysis of ChIP-Seq (MACS) peak-calling software for peak detection. Sequencing reads (BAM files) from both the ChIP sample and the corresponding control sample (e.g., input DNA) were aligned using Bowtie2. The MACS software was used to eliminate duplicate reads resulting from PCR amplification, ensuring only 1 read per identical genomic position in both the ChIP and control samples. Peaks were then identified through the MACS call peak function using a sliding window approach. The significance of each region was evaluated based on a *P* value derived from the Poisson model and lambda local, which refers to a local background signal estimation used to distinguish real binding sites from noise. Peaks surpassing a user-defined threshold (default was 5 × 10^–2^ [0.05]) were documented.

### Immunohistochemistry

Immunohistochemistry was performed on 5-μm-thick paraffin sections following the standardized protocol of Agarwal et al. (37). Briefly, slides were deparaffinized in 3 changes of xylene, 5 minutes each, followed by rehydration through a graded series of alcohol. Antigen retrieval was performed in citrate buffer (pH 6.0) for 10 minutes, followed by subsequent cooling for 20 minutes and blocking of endogenous peroxidase with 30% hydrogen peroxide. Slides were next incubated with the primary antibodies pSMAD2 (rabbit monoclonal pSMAD2 Ser-465/467 antibody, 1:50; Cell Signaling Technology) and ACVR2b (1:50; Mybiosource, 247275) at 4°C overnight, washed 3 times with Tris-buffered saline (5 minutes each), and incubated with biotinylated anti–rabbit secondary antibody (1:200) for 1 hour at room temperature. After treating the slides with HRP-conjugated ABC complex (Vectastain; Vector Laboratories) for 1 hour at room temperature; color was developed. DAB (Vector Laboratories) was counterstained with hematoxylin, mounted with DPX, and examined under an Olympus DP73 microscope for imaging, analysis, and interpretation. Sections from a reactive lymph node served as the positive control; those without the addition of a primary antibody served as the negative control.

### Cytokine arrays

After informed consent was given, blood samples were collected from patients for cytokine analysis and CFU assays. After centrifugation at 350 *g* for 15 minutes at 4°C, plasma was separated from whole blood. The supernatant was then collected and used for cytokine analysis and CFU assays. Cytokine analysis was outsourced to Raybiotech.

### Western blotting

For Western blotting, cells were harvested by centrifugation at 350 *g* for 10 minutes at 4°C and resuspended in 200 μL of lysis buffer (recipe). After 3 rounds of 5-second vortexing and 10-minute incubations on ice, the lysate was clarified by centrifugation at 10,000*g* for 15 minutes at 4°C. The resulting supernatant was collected, and protein concentration was quantified using the Bradford assay. We loaded 40 μg of protein onto 12% SDS-PAGE precast gels (Bio-Rad) and transferred these onto PVDF membranes using a Trans-Blot Turbo system (Bio-Rad). After blocking with Intercept (TBS) Blocking Buffer, the membranes were probed with primary and secondary antibodies, respectively. Immunoreactive protein bands were visualized using the LI-COR imaging system.

### CRISPR deletion of GATA1 regions

The CRISPR guide RNAs were designed using the Benchling website, targeting the specific genomic region of interest. The guide RNAs were then resuspended to a final concentration of 1.5 nmol/μL. For each transfection, 1.5 nmol of guide RNA was mixed with 0.8 μL of Cas9 enzyme and added to 700,000 cells. Electroporation was performed using the Lonza Nucleofector machine, following the manufacturer’s protocol. For HSC transfection, we used Protocol DZ-100; FF-120 was used for K562 cells. Five days after CRISPR guide-RNA electroporation, 100,000 cells were aliquoted for genomic DNA isolation to confirm the efficiency of CRISPR deletion.

### Flow cytometry

Human CFU sample colonies were counted after a 14-day incubation. The CFU cultures were resuspended in 2% FBS-PBS solution and incubated for 1 hour at 37°C. After incubation, the culture was thoroughly mixed, transferred into a 15 mL falcon tube, and the cells were pelleted at 350*g* for 10 minutes at 4°C. The cells were washed first with 2% FBS-PBS, followed by PBS alone. Cells were resuspended in 1:4000 diluted Zombie-NIR dye and incubated for 15 minutes. Cells were washed with PBS and resuspended in 50 μL of flow antibody cocktail. Cells were washed again in PBS and resuspended in 200 μL of PBS. Flow cytometry was then performed to acquire the data.

For FACS analysis, cells were suspended in FACS sorting buffer (PBS with 0.5% BSA and 2 mM EDTA) and further stained with indicated surface antibody markers for 30 minutes at 4°C. All flow cytometry data were acquired on a BD LSRII FACSymphony A3 Cell Analyzer and analyzed using FlowJo. To evaluate chimerism in transplantations, PBMCs were labeled using conjugated antibodies CD45.1-APC (BD Biosciences, 561873) and CD45.2-PerCP-Cy5.5 (BD Biosciences, 552950). Concurrently, erythroid cells underwent analysis through staining of unfractionated blood mononuclear cells (BMCs) with antibodies Ter119-APC (Biolegend, 116212) and CD44-V450 (BD Biosciences, 560452). Analysis of myeloerythroid progenitor cells was done by staining whole BMCs with a variety of conjugated antibodies: lineage cocktail V450 (BD Biosciences, 51-9006957); c-Kit-APC-eFluor780 (eBioscience, 47-1171-82); Sca-1-FITC (eBioscience, 11-5981-81); CD150-PE (Biolegend, 115904); CD41-PE-Cy7 (Biolegend, 113916); CD105-APC (eBioscience, 17-1051-82); and CD16/32-PerCP-Cy5.5 (BD Biosciences, 560540).

### Treatment of primary mice

As noted, sex typically is not considered a biological variable or a sex-specific trait in our studies. Male WT or *Gata1s* mice, aged 2 months, were administered either 10 mg/kg RAP-536 or PBS via intraperitoneal injection every Monday and Thursday for 8 weeks. Retro-orbital bleeding and complete blood cell count (CBC) analysis were performed weekly. Following the last injection, all mice were euthanized for bone marrow analysis in the subsequent week.

### Treatment of recipients from competitive bone marrow transplantation

*Gata1s* Mouse bone marrow cells (CD45.2^+^) were mixed with competitor bone marrow cells harvested from CD45.1^+^/CD45.2^+^ mice at a 1:1 ratio after lysing nucleated red blood cells. One million total bone marrow cells were then transplanted into lethally irradiated (1100 rad) CD45.1 recipient mice via tail vein injection. Recipient mice received either 10 mg/kg of RAP-536 or PBS via intraperitoneal injection every Monday and Thursday for 4 weeks. Retro-orbital bleeding, engraftment checking, and CBC analysis were performed weekly. After the last injection, all mice were euthanized and bone marrow analyzed in the subsequent week.

### Western blotting from mouse bone marrow

We isolated total BMCs from WT and *Gata1s* mice and cultured them under 2 different conditions. The first condition used an erythropoietin (EPO)-free medium (IMDM, 15% FBS, 10 μM β-ME, 1% pen-strep, 2 mM glutamine, 50 ng/mL stem cell factor, 30 ng/mL Flt-3L, 20 ng/mL IL-6); the second condition included EPO-containing medium (IMDM, 15% FBS, 1% BSA, 10 μg/mL human recombinant [rh] insulin, 200 μg/mL rh holo-transferrin, 10 μM β-ME, 1% pen-strep, 2 mM glutamine, and 2 U/mL EPO). We treated the cells with GDF-11 at a final concentration of 100 ng/mL and RAP-536 at 2 μg/mL for 12 hours. After treatment, we used an anti–mouse Ter-119 biotin antibody to separate the cells into 2 fractions using streptavidin magnetic beads: Ter119^+^ and Ter119^–^. Whole-cell lysates for Western blot analysis were prepared by adding RIPA buffer containing universal nuclease. Blots were probed with anti–mouse *GATA1* antibodies (Abcam, ab11852), and HSC70 was probed with anti–HSC70 antibodies (Santa Cruz, sc7298).

### Drug injections

At 24 hours after fertilization, embryos were dechorionated with the addition of pronase (Roche), anesthetized with tricaine, and then placed on an injection plate (1% agarose in a petri dish). A nonfilament microinjection needle was then trimmed and used for intravascular injection into the common cardinal vein. GDF11 (R&D Systems) at 1–1.5 ng or 1% BSA (Sigma-Aldrich) in Dulbecco’s PBS as a control was injected into all embryos, followed by a subsequent intravascular injection of 25–125 ng ACE536/luspatercept or 1% BSA control per embryo.

### O-dianisidine staining

*O*-dianisidine staining was performed in the manner described by Lieschke et al. (38). At 48 hours after fertilization (24 hours after drug injections), embryos were soaked in *o*-dianisidine solution (0.62 mg/mL *o*-dianisidine [Sigma-Aldrich], 10.9 μM sodium acetate, and 0.65% H_2_O_2_) in the dark for 10 minutes, then rinsed in phosphate-buffered solution with 0.1% Tween 20 3 times and preserved in 4% paraformaldehyde. Photographs were taken using a Zeiss Discovery V8 microscope, and staining was quantified with Image J software using the following protocol: the region of interest was traced manually around the edge of staining and quantified. Then, 3 background measurements were taken and the mean intensities were averaged. A list of reagents used in this study is given in Tables 1 and 2.

Quantification was calculated as the raw integrated density of the region of interest minus the area of the region of interest multiplied by the average of the background means. The Mann-Whitney test was used to calculate the difference between the *o*-dianisidine staining quantifications of different groups.

### Calculation of cell doubling times

Cell doubling times were calculated using an online resource (https://www.omnicalculator.com/biology/cell-doubling-time).

### Statistics

Unless stated otherwise, mean values with SDs are presented throughout. A *P* value of less than 0.05 was considered statistically significant. Non-normally distributed data were analyzed using the Mann-Whitney test. Immunoblot image quantitation was based on at least 3 independent biological replicates. Data visualization was done with GraphPad Prism, version 8 software.

### Study approval

All fish were maintained according to IACUC-approved protocols in accordance with Albert Einstein College of Medicine research guidelines. WT embryos were of AB genetic background. Animal studies (mouse) were approved by St. Jude Children’s Research Hospital. Our previous study (19) demonstrated no discernible difference in erythropoiesis defects between *Gata1s* male mice and *Gata1s* female mice from fetal development to adulthood. We did observe that *Gata1s* female mice exhibited heightened sensitivity to phenylhydrazine upon the introduction of an acute hemolytic anemia (2 died). This finding was primarily attributed to the ease of generating male *Gata1s* hemizygous mice compared with female *Gata1s* homozygous mice.

Patients signed informed consent in accordance with the Declaration of Helsinki, and the study received approval from the Albert Einstein College of Medicine Institutional Review Boards.

### Biological material and data availability

For information and requests for reagents, please contact AV. All the resources, including RNA-Seq and ChIP-Seq data, have been deposited in the GEO database (accession nos. GSE292039 and GE292213, respectively). Supporting data values are provided along with the manuscript.

## Author contributions

SA, AV, SV, and JDC conceptualized the study. SA, AV, SV, JDC, TVB, RNVSS, JB, US, SN, AS, and AW contributed to the study methodology. SA, TL, EF, SC, KZ, AA, GP, SS, RZ, AC, KB, JIY, AB, BA, SGM, MC, and AP conducted the investigation. SA, TL, EF, KP, SA, LK, AV, SV, RS, TVB, and JDC conducted the data analysis. SA, TL, EF, and AV wrote the manuscript. SA and AV reviewed and edited the manuscript. AV, SV, and JDC supervised the study. AV, SV, RS, and JDC contributed to funding acquisition.

## Supplementary Material

Supplemental data

Unedited blot and gel images

Supplemental table 1

Supporting data values

## Figures and Tables

**Table 1:**
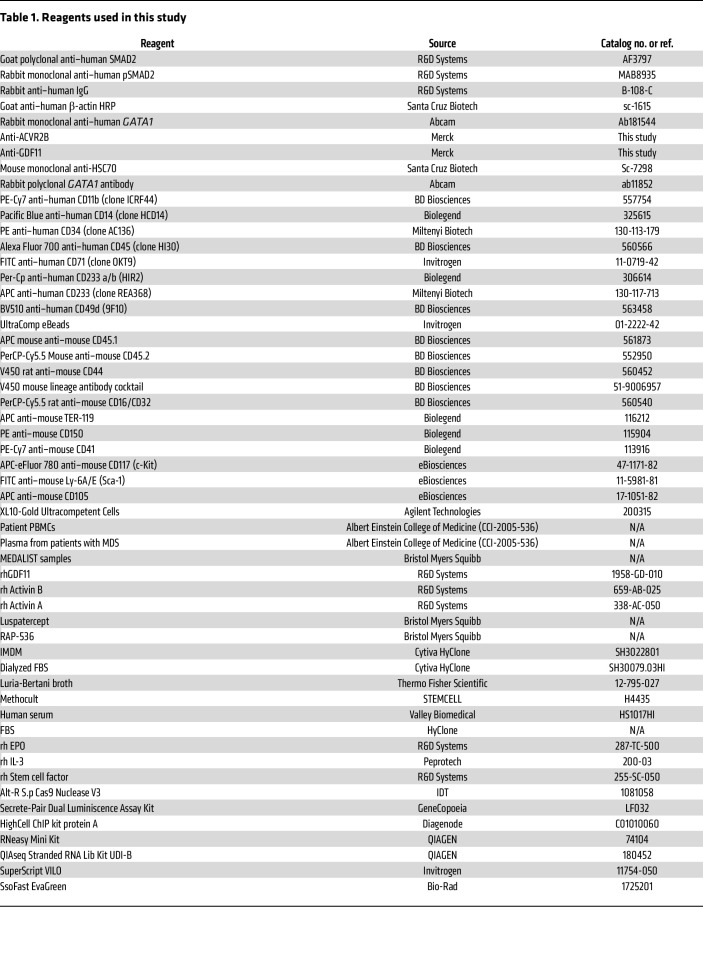
Reagents used in this study

**Table 2:**
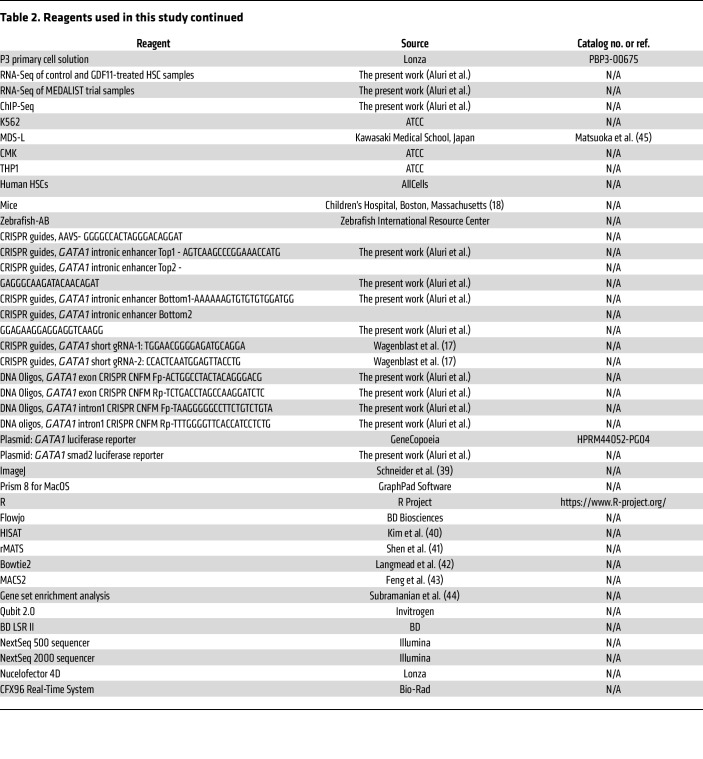
Reagents used in this study continued

## Version 1 05/27/2025

In-Press Preview

## Version 2 07/01/2025

Electronic publication
