# Supplementary material for: Splicing of erythroid transcription factor is associated with therapeutic response in myelodysplastic syndromes
Source: J Clin Invest. 2025 May 27;135(13):e189266. doi: 10.1172/JCI189266 (PMC12208544; doi:10.1172/JCI189266)
Supplement: Supplemental data [file jci-135-189266-s135.pdf]

Figure S1

A

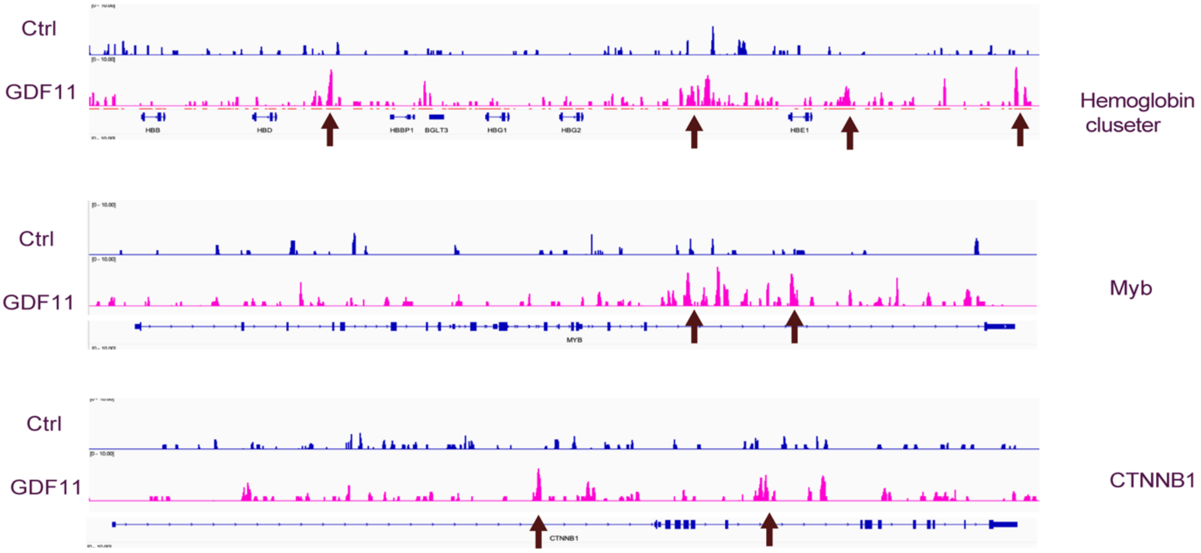

B

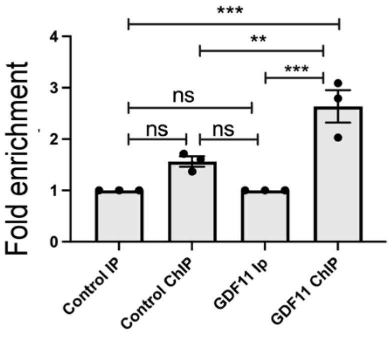

Figure S1. (A) IGV plots showing SMAD2 peaks observed at multiple gene loci. SMAD2-bound regions are indicated by an arrow. (B) qPCR bar plot showing the confirmation of a SMAD2-bound peak in the GATA1 intron. Control Ip: Control input; Chip Ip: ChIP input. * *P < 0.05,* ANOVA*.*

Figure S2

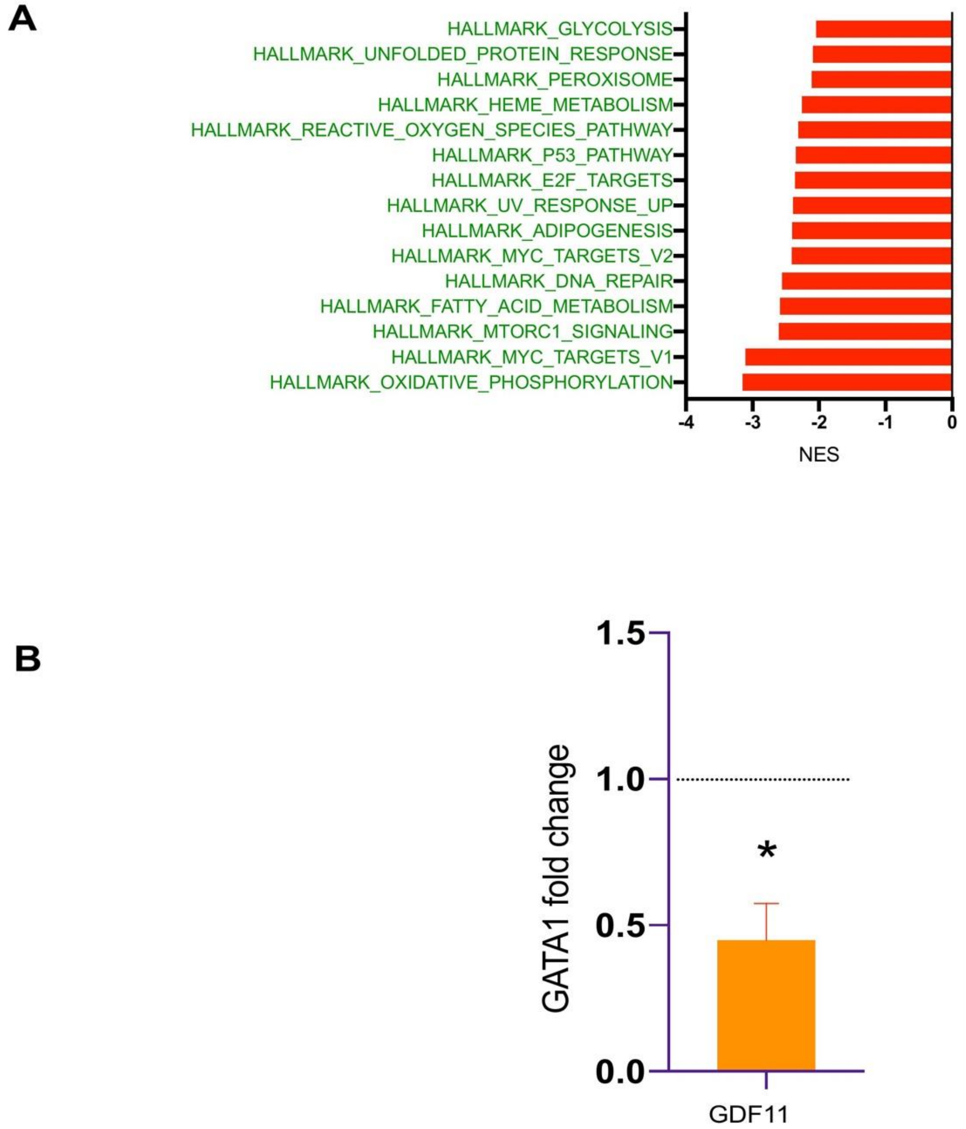

Figure S2. (A) GSEA HALLMARK pathways are downregulated in GDF11 treated erythroid progenitors. (B) GATA1 RNA quantification from RNA-seq data from erythroid progenitors treated with GDF11 compared to control. * *P < 0.05,* Student's t-test.

Figure S3

A

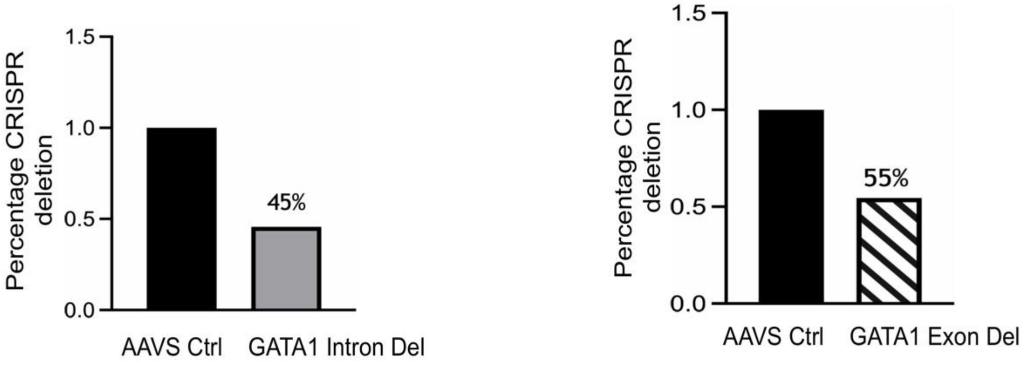

B

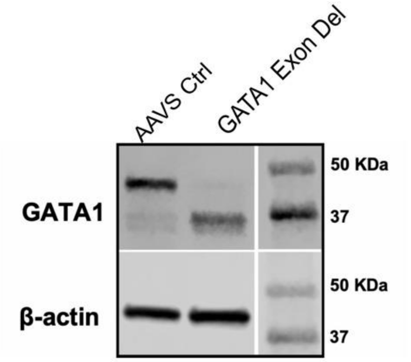

Figure S3. (A) qPCR plot showing the fold change up on CRISPR deletion of Intron 1 of GATA1 (left) and GATA1 exon (right). (B) Western blot showing the expression of GATA1 in Exon2 CRISPR/Cas9-edited cells. (C) DNA sequence showing pSMAD2 binding at the GATA1 intronic region. The putative pSMAD2 binding is highlighted in yellow. The CRISPR guideRNA sequence is highlighted in bold.

Figure S4

A

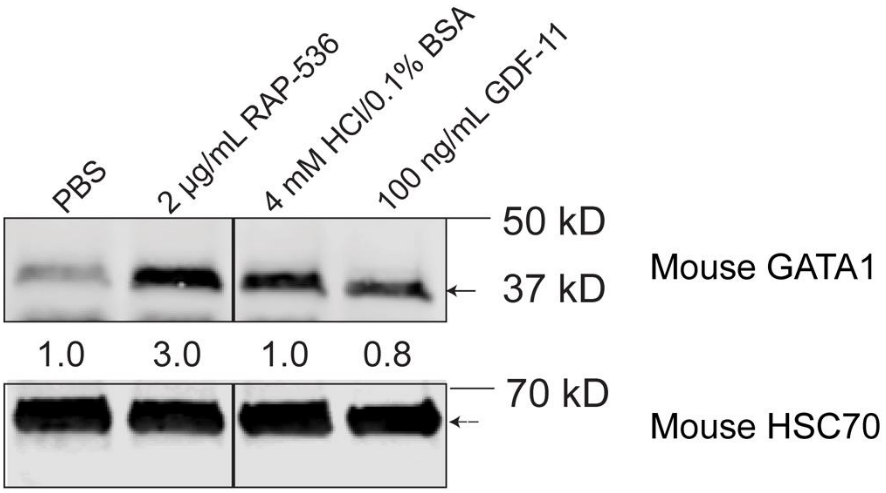

Figure S4. (A) Representative western blot images for mouse *gata1* expression. BMCs from *gata1s* mouse were treated with GDF11, vehicle or a combination of RAP-536 for 12 h and then assessed for the expression of *gata1*.

Figure S5

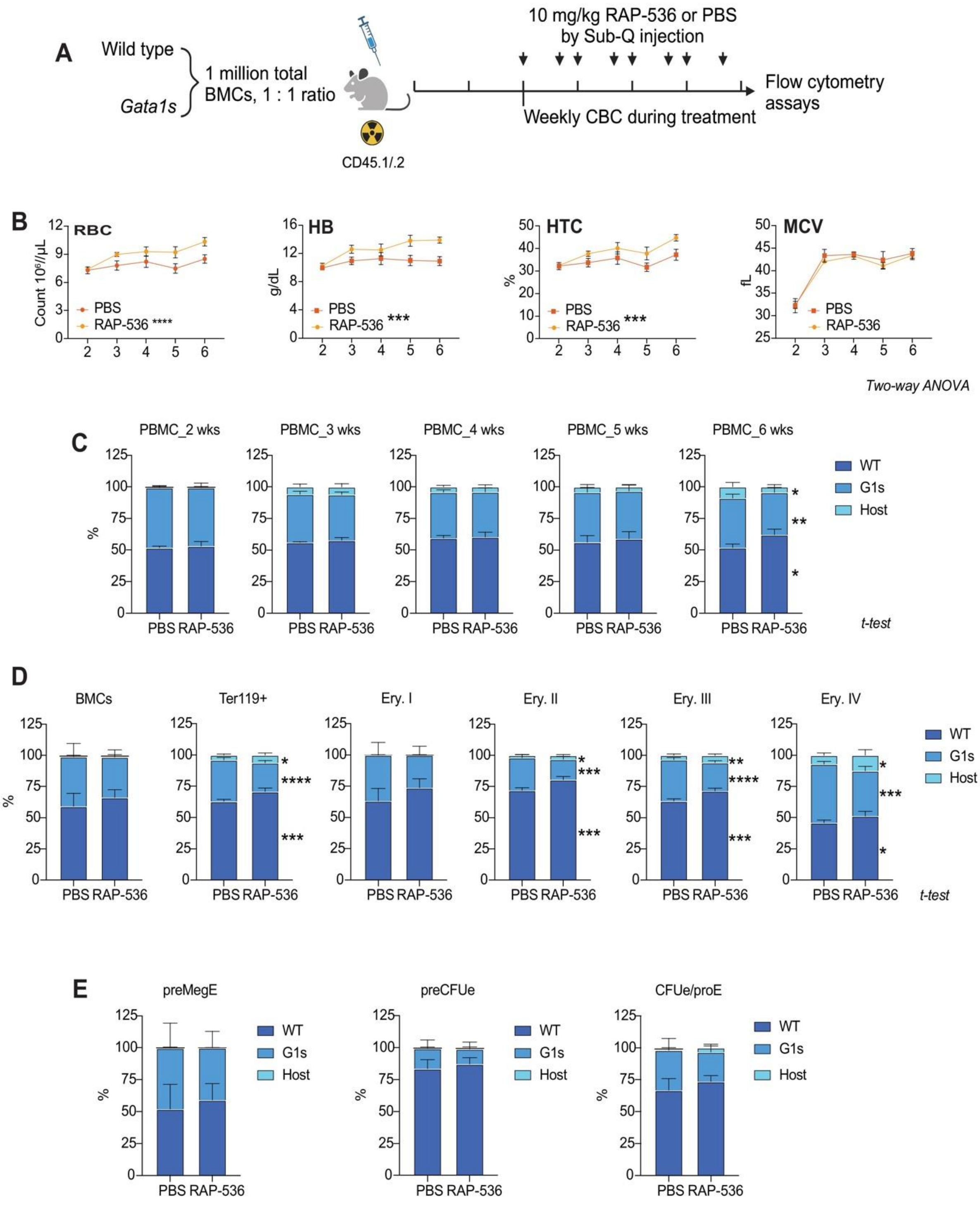

Figure S5. A) Experimental schema. 500,000 wild-type and 500,000 *Gata1s* bone marrow cells were combined and engrafted into irradiated recipient mice. These animals were treated with RAP-536 or vehicle for four weeks and bled weekly to monitor peripheral blood counts. Mice were euthanized at the end of the study for flow cytometry analysis of the bone marrow. B) Peripheral blood counts of the mice during the course of therapy. *** *p<0.001;* **** *p<0.0001,* Two-way Anova. C) Comparison of the percentages of WT and *Gata1s* donor populations in the peripheral blood during the course of the study. Host cells represent those cells that remain after irradiation. *p<0.05;* ** *p<0.01,* Student's t-test. D) Comparison of the percentages of WT or *Gata1s* engrafted cells in the bone marrow of recipient mice at the endpoint. Host cells represent those cells that remain after irradiation. * *p<0.05;* ** *p<0.01;* *** *p<0.001;* **** *p<0.0001,* Student's t-test. E) Comparison of the percentages of WT or *Gata1s* engrafted progenitor cell populations in the bone marrow of recipient mice at the endpoint. Host cells represent those cells that remain after irradiation. The mean +/- standard deviations are shown. RBC, red blood cell count; HB, hemoglobin; HCT, hematocrit; MCV, mean corpuscular volume; PBMC, peripheral blood mononuclear cells.

Figure S6

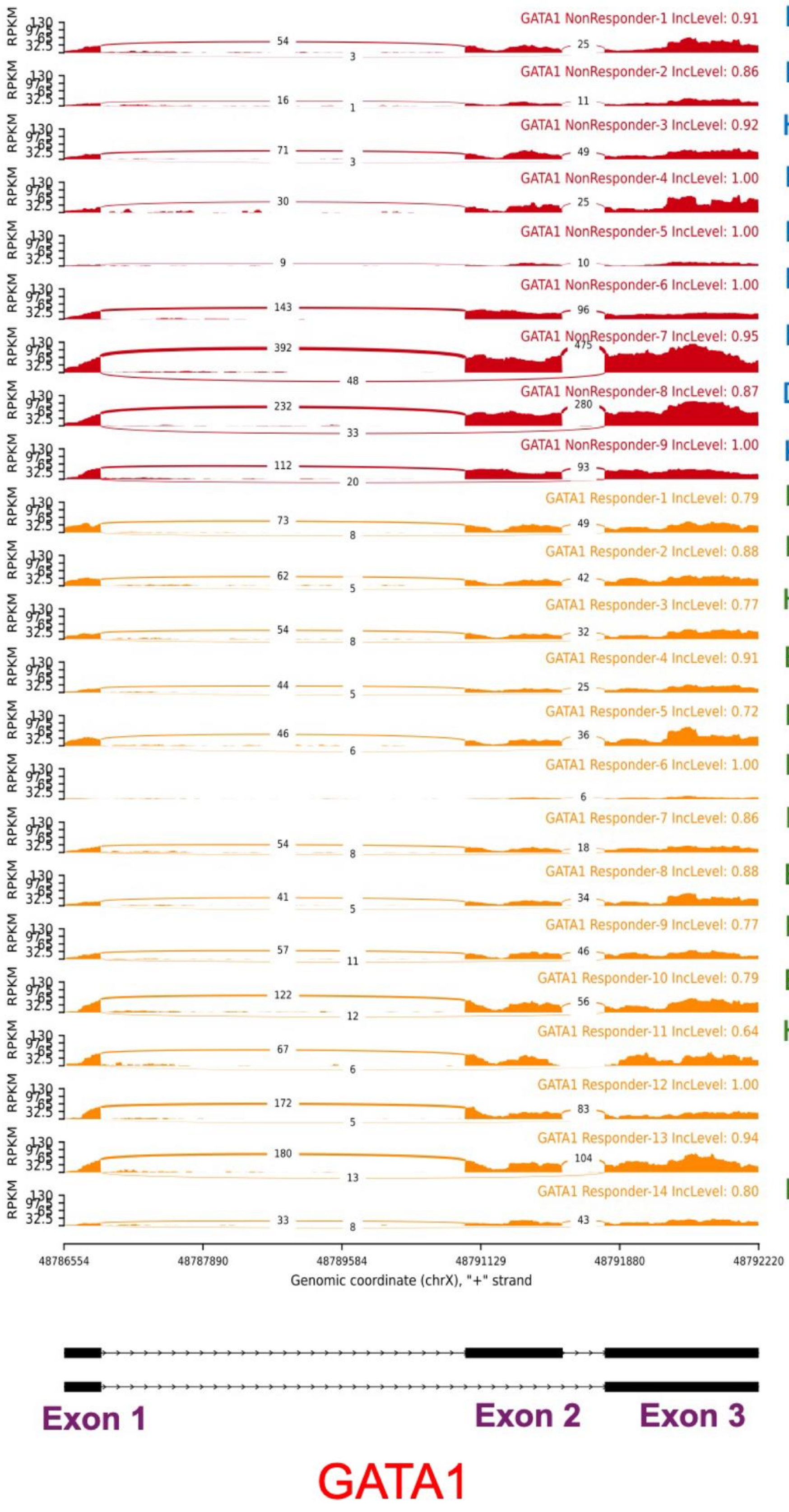

Sf3b1	Hb C1D1	Hb Week 24
K700E	7.3	7.3
K700E	9.7	10.7
H662Q	9.4	9.7
K700E	9.1	11.2
K700E	10.5	9.2
K700E	8.5	7.8
K700E	10.1	9.6
D781G	8.9	7.1
K666R	9.6	9.7
K700E	10	8.6
K700E	9.1	8.1
H662Q	8.6	10.2
E622D	9.5	9.7
K700E	9	10.6
K700E	7.2	8.8
L773R	7.3	9.2
E622D	10.2	10.7
K700E	9	10.6
E622D	10.2	10.7
H662Q	7.8	9.7
wt	8.2	9.8
H662Q, K700E	8.3	NA
K700E	9.5	11.6

Figure S6. Sashimi plot images for GATA1 mRNA splicing from BMCs of MEDALIST trial patients. Responders (orange) and non-responders (red) sashimi plots are showm along with IncLevels (inclusion level of exon 2). On the right respective *sf3b1* mutations in these patients are shown along with day 1 and week 24 hemoglobin values.
