# Supplementary material for: Splicing of erythroid transcription factor is associated with therapeutic response in myelodysplastic syndromes
Source: J Clin Invest. 2025 May 27;135(13):e189266. doi: 10.1172/JCI189266 (PMC12208544; doi:10.1172/JCI189266)
Supplement: Unedited blot and gel images [file jci-135-189266-s136.pdf]

Fig 2B

MDS-L

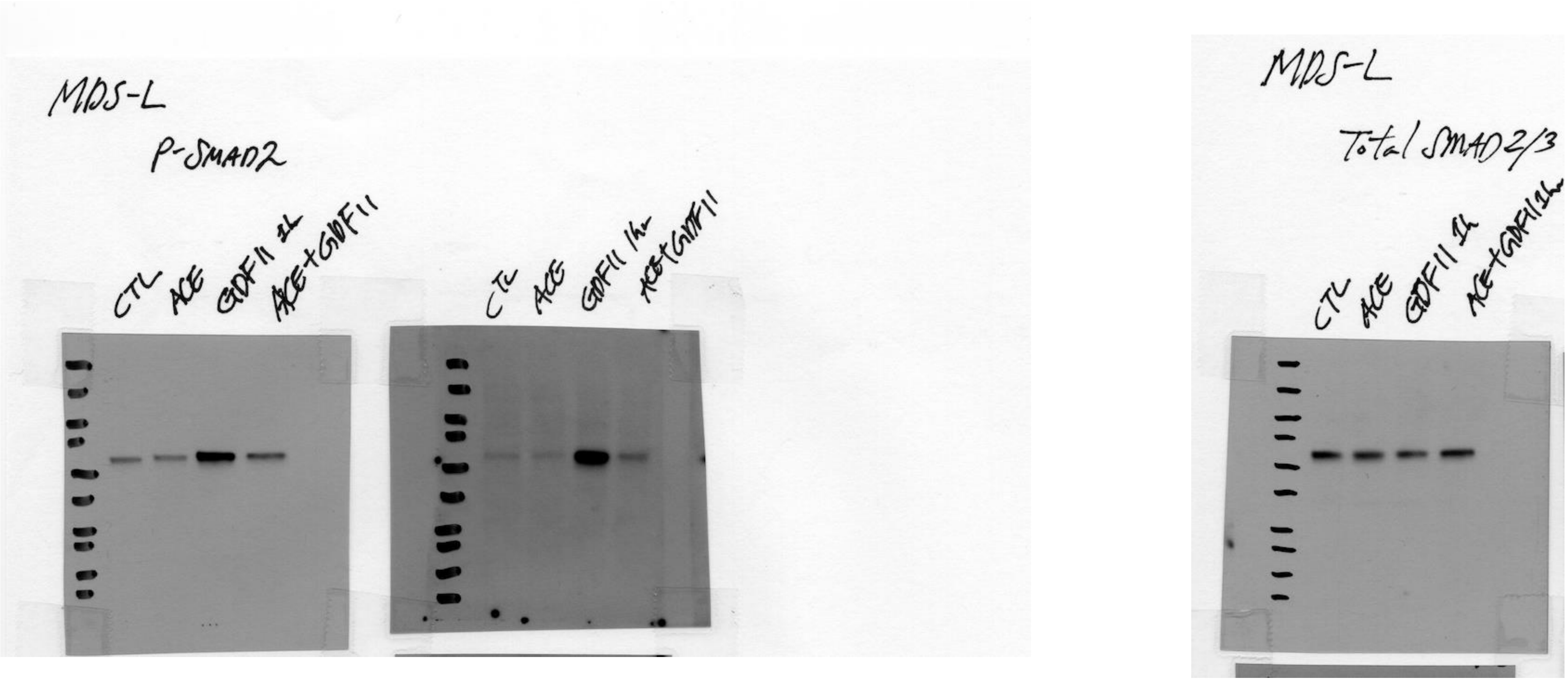
MDS-L
P-SMAD2
CTL
ACE
ACE+GDF11
CTL
ACE
ACE+GDF11
MDS-L
Total SMAD2/3
CTL
ACE

Fig 2B **CD34+**

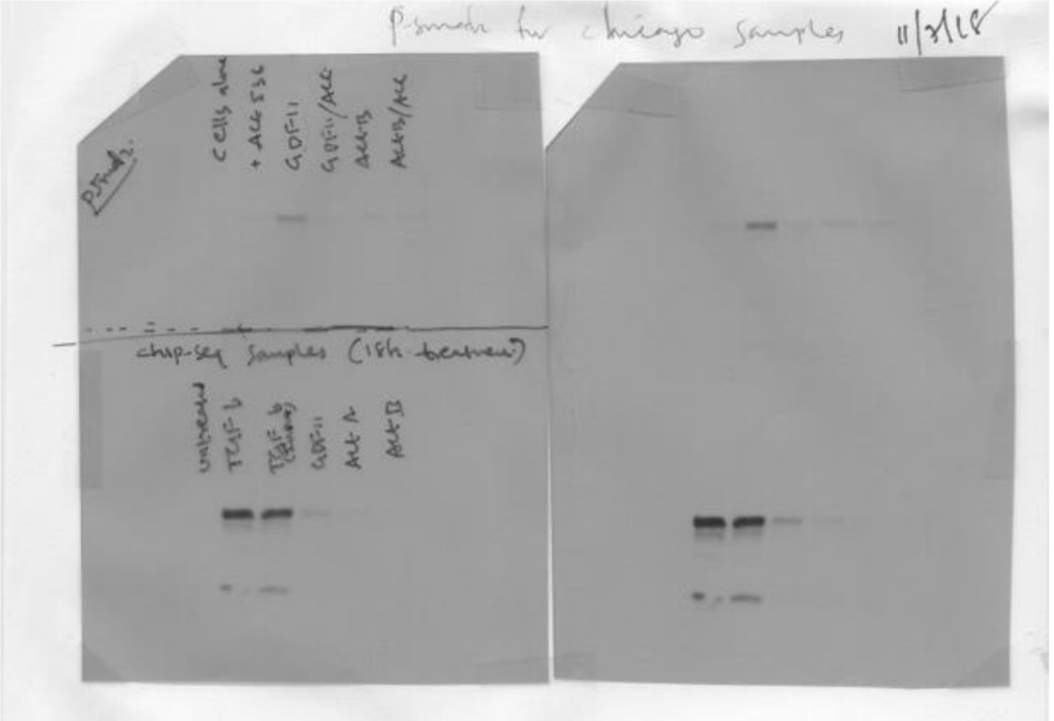
chip-seq samples (18h treatment)
GDF11
Act A
Act B

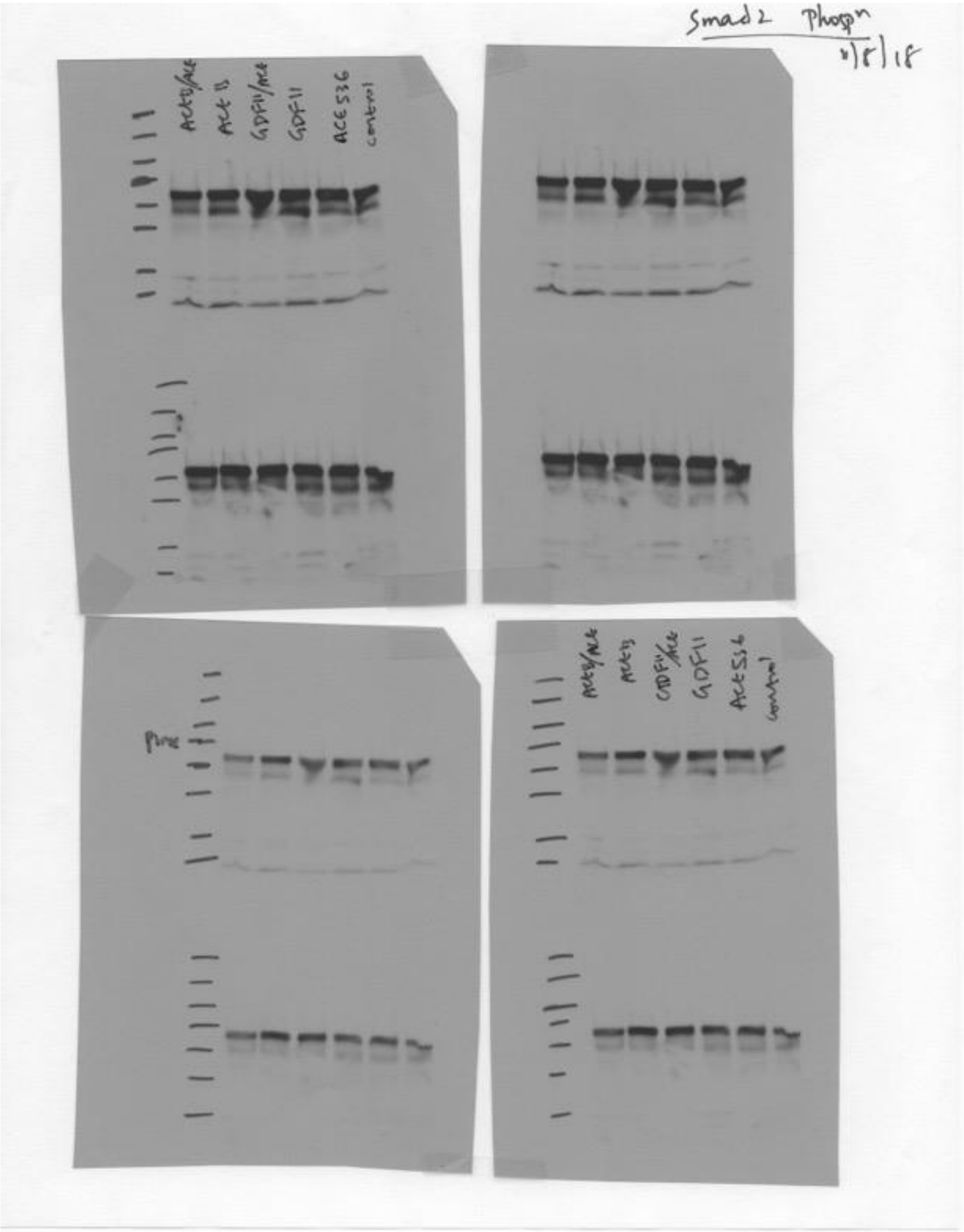
Smad2 Phosph
Act B
GDF11
control

Fig 2B

CMK

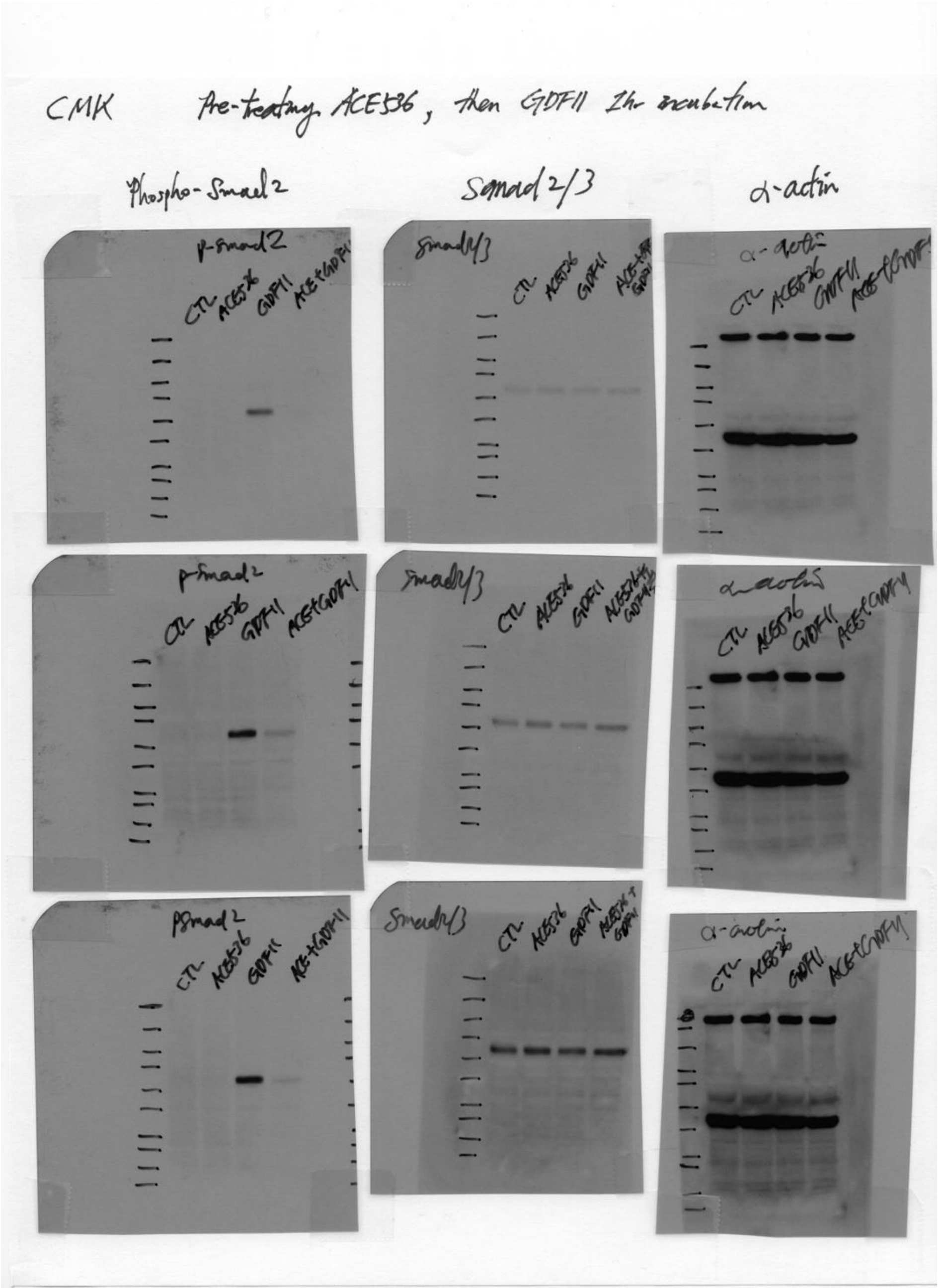
CMK Pre-treating ACE536, then GDF11 1hr incubation
Phospho-Smad2
Smad2/3
α-actin
p-Smad2
CTL
ACE536
GDF11
ACE+GDF11
Smad2/3
α-actin
pSmad2

Fig 2B **THP1**

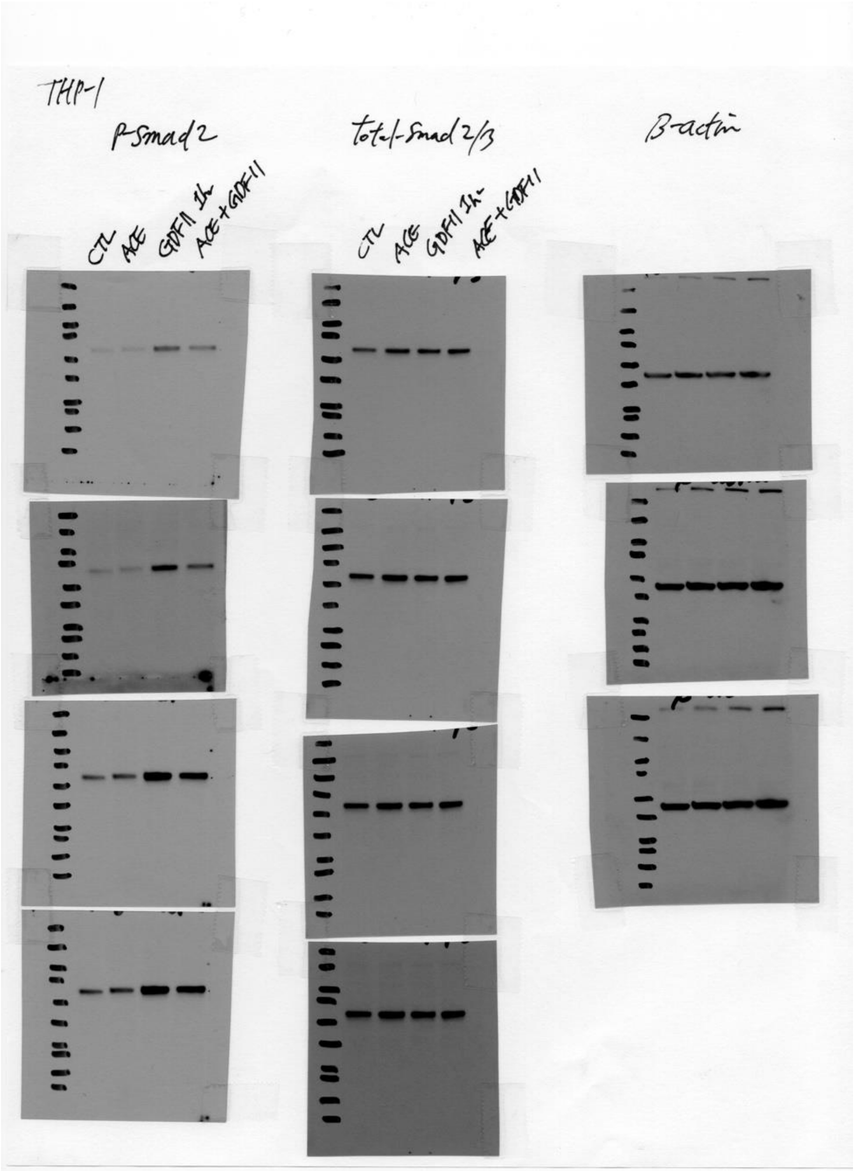

Fig 3I

Lane1: CD34+ Ctrl
Lane2: CD34+ GDF11
Lane3: CD34+ GDF11-Luspa

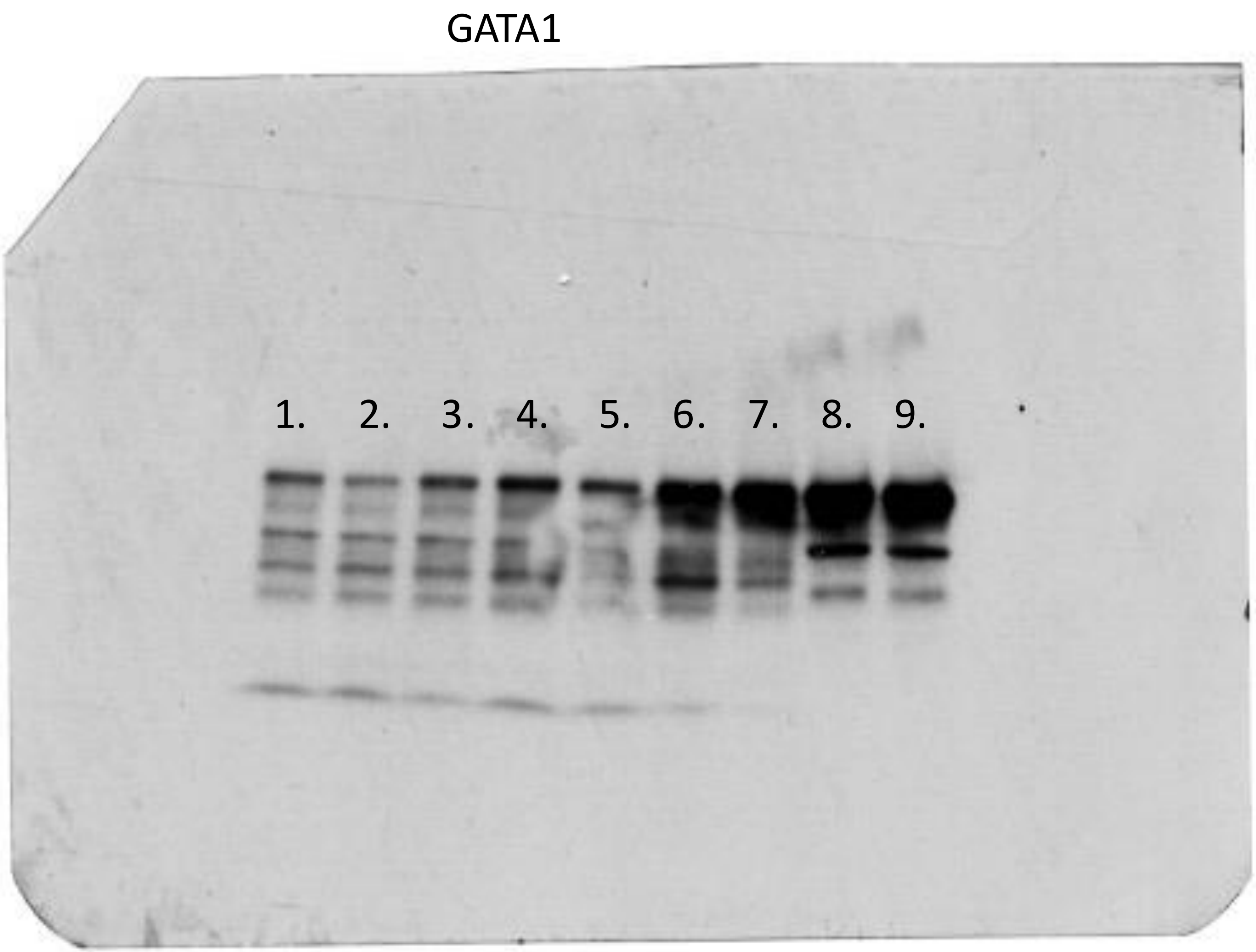

Lane 4: AAVS
Lane 5: G1G2

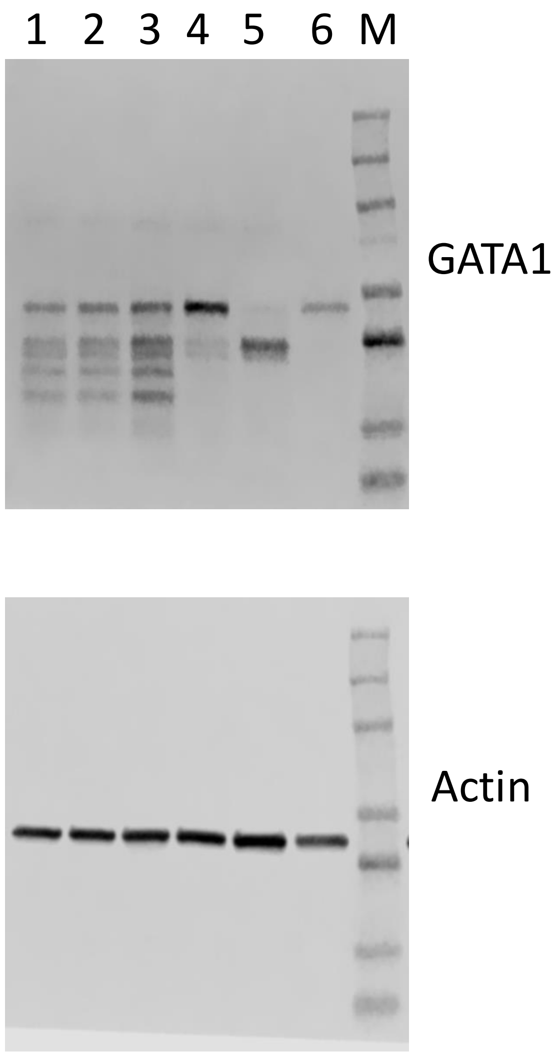

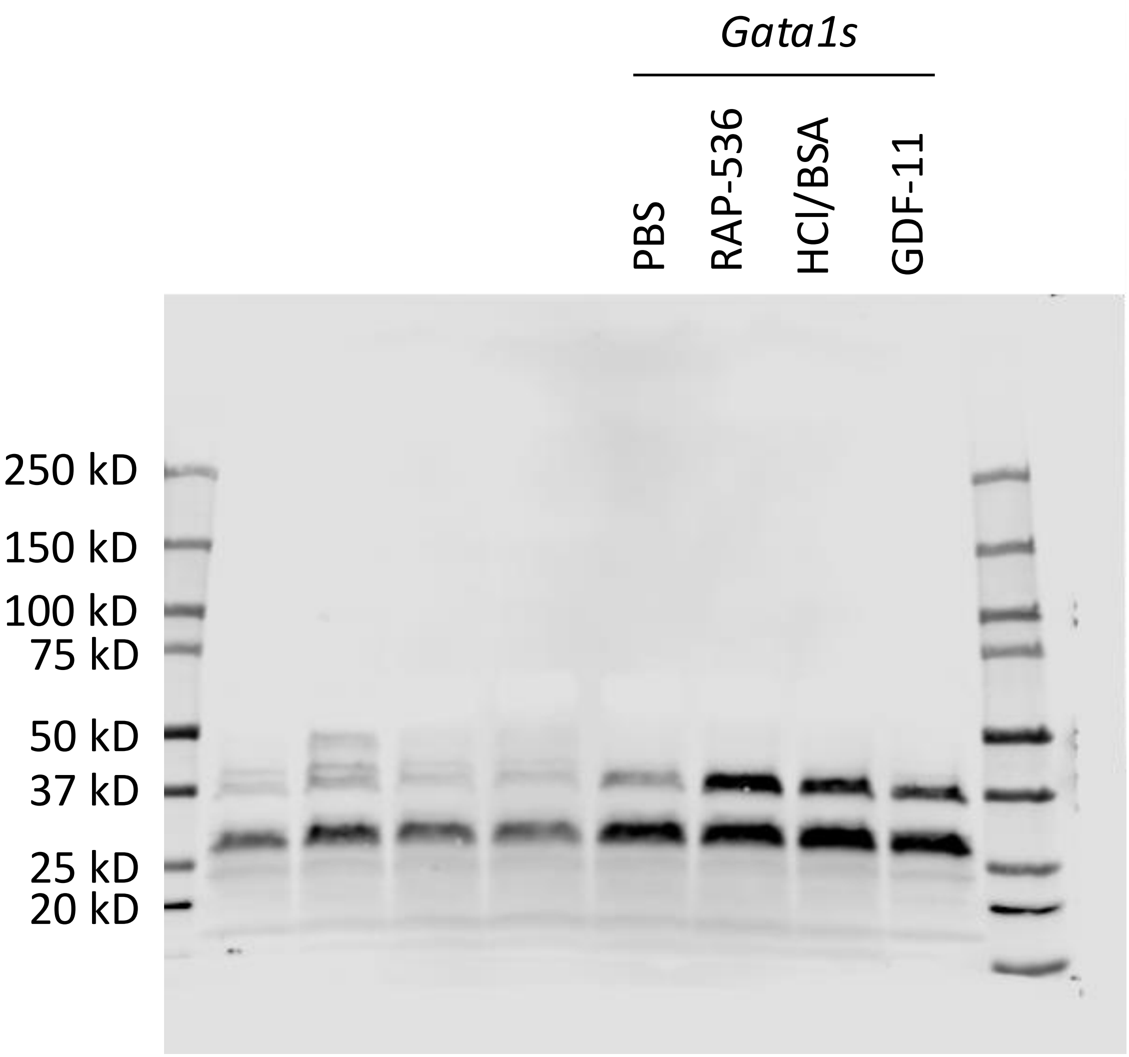
Gata1s
PBS
RAP-536
HCl/BSA
GDF-11
250 kD
150 kD
100 kD
75 kD
50 kD
37 kD
25 kD
20 kD
